# Reduced dosage of *Kmt2d* modifies *Tbx1* haploinsufficiency toward phenotypes of 22q11.2DS

**DOI:** 10.1172/jci.insight.206763

**Published:** 2026-08-24

**Authors:** Daniella Miller, Kevyn Jackson, Timothy C. Cox, Bernice E. Morrow

**Affiliations:** 1 Department of Genetics, Department of Obstetrics & Gynecology and Women’s Health, and Department of Pediatrics, Albert Einstein College of Medicine, Bronx, New York, USA.; 2 Department of Oral and Craniofacial Sciences and Department of Pediatrics, University of Missouri–Kansas City, Kansas City, Missouri, USA.

**Keywords:** Cardiology, Development, Genetics, Embryonic development, Genetic diseases, Mouse models

## Abstract

Haploinsufficiency of *TBX1*, which occurs in 22q11.2 deletion syndrome (22q11.2DS), leads to a heterogeneous spectrum of clinical manifestations, including craniofacial anomalies, immunodeficiency, and congenital heart defects. The variability in syndromic presentation between patients may be partially explained by variants in chromatin regulatory genes that act to further modify TBX1 function. To investigate this relationship, we selected *KMT2D* as a candidate gene because of its role in the etiology of Kabuki syndrome, which shares overlapping features with 22q11.2DS. We demonstrate that conditional inactivation of *Kmt2d* in the *Tbx1* lineage in *Tbx1*-heterozygous mice leads to fully penetrant perinatal lethality and increased incidence of craniofacial dysmorphism, thymus and parathyroid gland hypoplasia, and aortic arch anomalies. At early stages, mutant embryos were found to have defects of the caudal pharyngeal apparatus, including abnormal patterning of the third pouch endoderm, hypoplastic fourth arches, and defective fourth arch arteries. Finally, analysis of single-cell RNA sequencing revealed dysregulation, and largely downregulation, of genes involved in basic cellular functions, suggesting that *Tbx1* and *Kmt2d* developmentally converge upon essential biological processes. Overall, these results indicate that reduced dosage of *Kmt2d* perturbs the developmental landscape of the *Tbx1* heterozygote, eliciting phenotypes that are shared between 22q11.2DS and Kabuki syndrome.

## Abstract 2

Using mouse models we have uncovered a novel relationship between *Tbx1*, implicated in 22q11.2 deletion syndrome, and *Kmt2d*, the causative gene for Kabuki syndrome.

## Graphical abstract

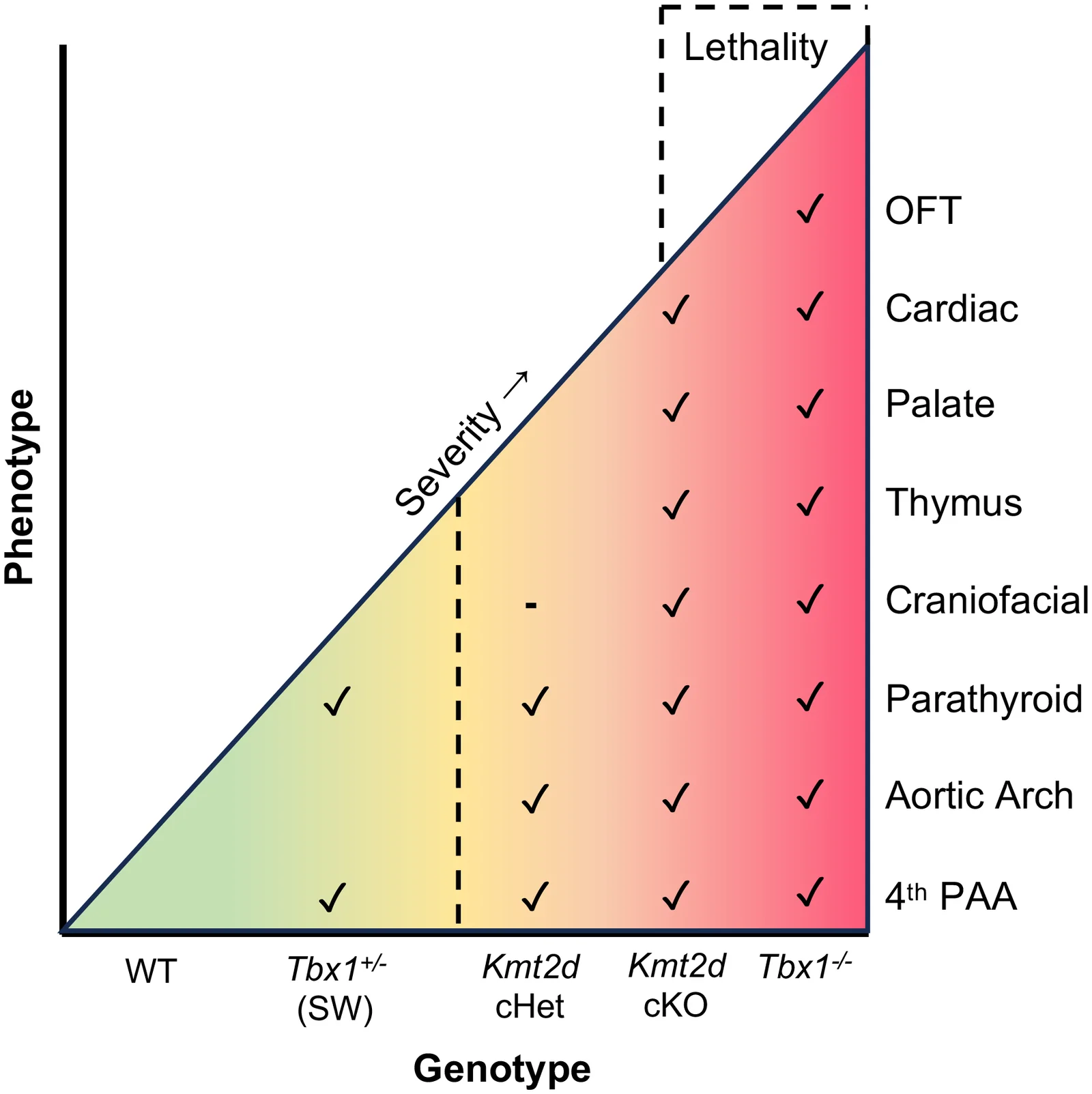

## Introduction

In 22q11.2 deletion syndrome (22q11.2DS), which occurs in 1 in 4,000 live births, around 90% of patients present with an approximately 3 million-base-pair hemizygous deletion on chromosome 22q11.2 (1). Despite this similarity in deletion size, there is high variability in the clinical presentation, which can include characteristic craniofacial features, palatal abnormalities, immunodeficiency, hypoparathyroidism, and cardiac anomalies, among others (1). Although 22q11.2DS occurs with a hemizygous deletion of more than 45 protein-coding genes (2), a main gene responsible for cardiac and other clinical manifestations is *TBX1*, which encodes a T-box transcription factor whose function is sensitive to gene dosage. The classic cardiac presentation involves the outflow tract (OFT), leading to conotruncal defects (CTDs) that include tetralogy of Fallot, retroesophageal right subclavian artery (RERSA), interrupted aortic arch type B (IAAB), and persistent truncus arteriosus (PTA) (3); many of these defects cause early mortality in 22q11.2DS, although they only occur in about 60%–80% of patients. The basis of variable phenotypic expression is a long-studied question with several explanatory theories that likely exist in concert, including the occurrence of stochastic developmental events during embryogenesis, differences in environmental exposure in utero, and genetic modifiers on the remaining allele of 22q11.2 or elsewhere in the genome (2). In this study, we attempt to expound on the role of genetic modifiers in *TBX1* phenotypes, as they are readily identifiable with current technologies and may eventually represent targets for future therapeutic intervention (4).

To identify possible modifiers, short-read whole-genome sequencing was previously performed on a cohort of 1,182 subjects with 22q11.2DS who were stratified by presence of CTDs versus those with normal cardiac anatomy (5). This revealed rare, potentially deleterious coding or splicing DNA variants in chromatin regulatory genes that occurred in 8.5% of the 22q11.2DS cases with CTDs (5). In independent studies of sporadic congenital heart disease, de novo mutations were found in some of the same chromatin regulatory genes, including *KMT2C*, *KAT6A*, *NSD1*, and *KMT2D* (6). Mouse genetic studies have shed some light on how *Tbx1* might interact with chromatin regulatory genes in various developmental pathways (7–9); our work here aims to expand the known regulatory network of the 22q11.2DS gene *Tbx1* in mouse models, with a particular focus on *Kmt2d*. *KMT2D*, the main causative gene in Kabuki syndrome, functions as an H3K4 methyltransferase that modifies histones at enhancer regions in order to activate gene expression (10). Kabuki syndrome has many overlapping features with 22q11.2DS, including cardiac defects (a subset of which are CTDs), palatal defects, craniofacial anomalies, and developmental delay (11, 12). Thus, while each of the chromatin genes identified in our previous study is of interest for further exploration, *Kmt2d* was particularly compelling to investigate with respect to the function of *Tbx1*.

*Tbx1* has a dosage sensitivity directly related to severity of phenotype (13–15), with anomalies in mouse models mirroring those on the 22q11.2DS spectrum (16). *Tbx1*-heterozygous mice have hypoplastic or absent fourth pharyngeal arch arteries (PAAs) at variable penetrance depending on genetic background, but largely undergo recovery and are otherwise viable (17, 18). *Tbx1-*null mutant mice, in which both copies of the gene are inactivated, die at birth with an overt cleft palate, absent parathyroid and thymus glands, and a PTA that results from failure of OFT septation (18). The structures impacted in *Tbx1*-mutant mouse embryos are largely derived from the pharyngeal apparatus, a transient developmental structure that is composed of ectoderm, endoderm, mesoderm, and neural crest cells (NCCs) (19–21). *Tbx1* is expressed in all three germ layers of the pharyngeal apparatus and has a non-autonomous role in NCC function; thus, *Tbx1*-null embryos fail to form pharyngeal arches 2–6, explaining the spectrum of phenotypes described above (22, 23). Our interest is to clarify whether and how *Kmt2d* cooperates with *Tbx1* in pharyngeal apparatus development, potentially highlighting a mechanistic overlap between the two genes that underlies aspects of their shared phenotypes. To do this, we inactivated a floxed allele of *Kmt2d* using the knockin *Tbx1^Cre/+^* allele that also functions as a *Tbx1* heterozygote (24). By inactivating either one or both copies of *Kmt2d* in this lineage, we are modeling the rare patients from our cohort who have variants in *KMT2D* along with *TBX1* haploinsufficiency (5). It is important to note that the KMT2D variants found in the 22q11.2DS patient cohort were missense variants of uncertain significance; we theorize that, while they may not be pathogenic in isolation, they have a synergistic effect when combined with the 22q11.2 deletion. Our results reveal dose-sensitive developmental roles of *Kmt2d* in the morphogenesis of *Tbx1*-dependent tissues, as evidenced by mild defects when one copy is conditionally deleted, and more severe defects when both are conditionally inactivated. Finally, single-cell transcriptomic analysis reveals downregulation of genes involved in transcription, cell cycle, and various signaling pathways, suggesting that *Kmt2d* impacts the developmental landscape of the *Tbx1* heterozygote through shared essential pathways.

## Results

### Conditional inactivation of Kmt2d in the Tbx1 lineage results in perinatal lethality with mild craniofacial dysmorphism and feeding defects.

**Figure 1:**
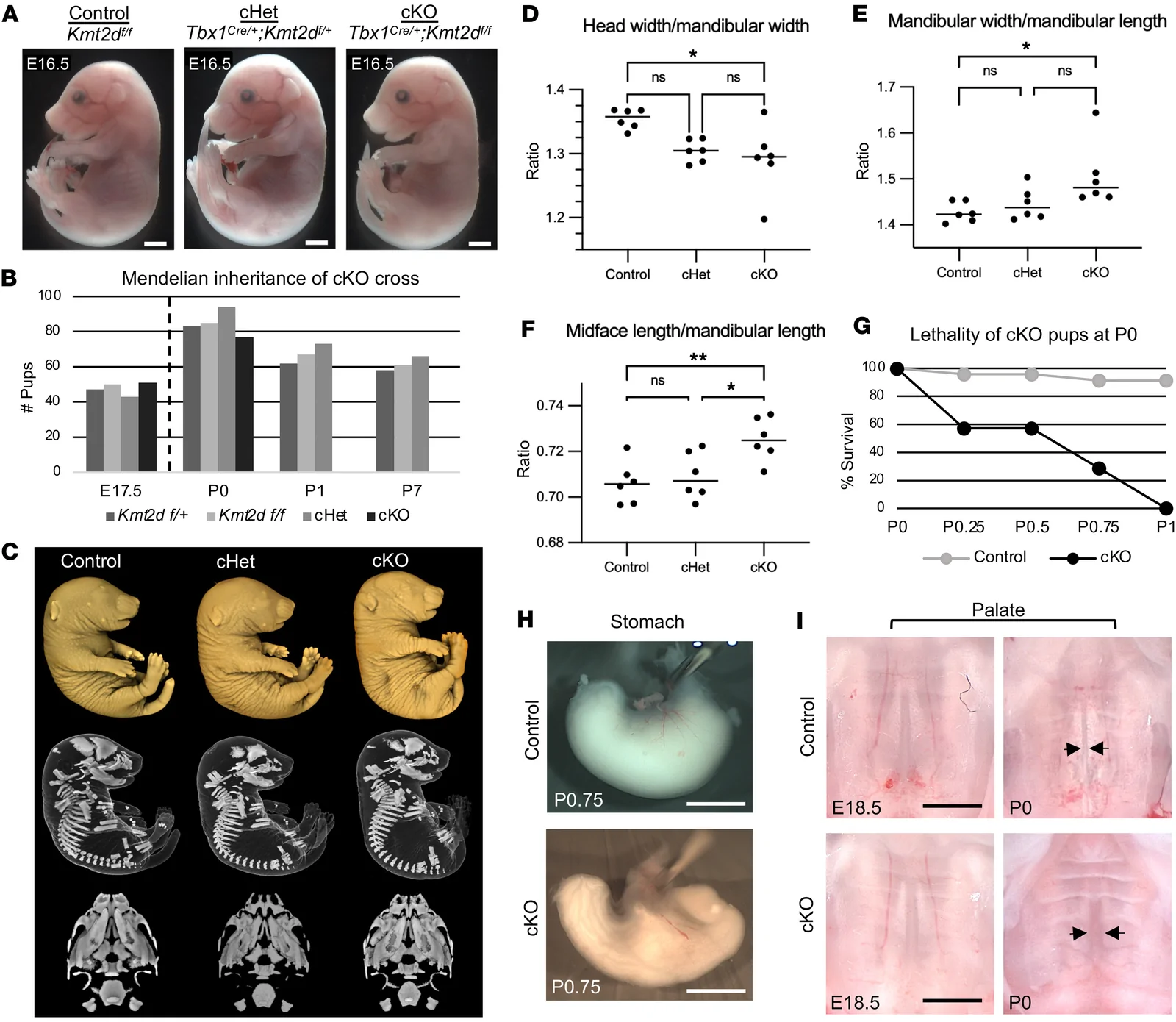
Inactivation of *Kmt2d* in the *Tbx1* lineage leads to fully penetrant perinatal lethality. (**A**) Embryos at E16.5 show no gross morphological defects (*n* = >10 per genotype). (**B**) There was no difference in Mendelian ratios between control (*Kmt2d^fl/+^*, *Kmt2d^fl/fl^*; without *Cre*), cHet, and cKO embryos in utero (E17.5, *n* = 191) or at time of birth (P0, *n* = 339). Dashed line separates fetal from neonatal life. P0, P1, and P7 genotypes represent sequential data from the same litters. All cKO pups died by P1, and no significant difference in survival was found in the remaining genotypes at P7. (**C**) Micro-CT scans of embryos at E17.5 showing full-body scans, skeletal structure, and cranial bones of the different genotypes of interest (*n* = 6). (**D**) Graph of head width/mandibular width showing a significant decrease in cKO compared with control embryos (*n* = 6 per genotype). (**E**) Graph of mandibular width/mandibular length showing a significant increase in cKO embryos compared with controls (*n* = 6 per genotype). (**F**) Graph of midface length/mandibular length showing a significant increase in cKO embryos compared with both controls and cHets (*n* = 6 per genotype). (**G**) Lethality curve showing time of death in control (*n* = 23) versus cKO (*n* = 21) pups over the course of P0 in 6-hour intervals. (**H**) Control stomach at P0.75 showing presence of milk as compared with cKO embryos, which had absence of milk (*n* = 8 for both genotypes). (**I**) Palates of control (*n* = 8) and cKO (*n* = 7) embryos at E18.5 and P0 show reduced closure of the posterior palate in cKO embryos and neonates. Scale bars: (**A**) 2 mm, (**H**) 5 mm, (**I**) 1 mm. *P < 0.05; **P < 0.01. Statistical analysis was performed using 3-way ANOVA.

We began by validating that the *Kmt2d-*floxed allele functions as previously described by inactivating it in the *Mesp1^Cre^* lineage and recapitulating previous findings of early lethality by embryonic day 10.5 (E10.5) (25) (Supplemental Figure 1, A–C; supplemental material available online with this article; https://doi.org/10.1172/jci.insight.206763DS1). We then set out to determine whether *Kmt2d* and *Tbx1* genetically interact by conditionally inactivating *Kmt2d* in the *Tbx1* lineage and assessing for embryonic or perinatal lethality. *Tbx1^Cre/+^* mice have a knockin of the *Cre* gene to the coding region of exon 5, therefore functioning as *Tbx1* heterozygotes (26). *Tbx1^Cre^* mice were crossed with homozygous *Kmt2d*-floxed mice (*Kmt2d^fl/fl^*) (25) to test whether there are shared and/or separate functions between *Tbx1* and *Kmt2d*, in the *Tbx1* lineage. Analysis of the Mendelian ratio of live pups at weaning age (postnatal day 21 [P21]) showed no significant difference in the number of *Tbx1^Cre/+^ Kmt2d^fl/+^* (conditional heterozygote [cHet]) mice as compared with control *Kmt2d^fl/+^* mice in a mixed genetic background (Supplemental Figure 1D). The cHets were then crossed with *Kmt2d^fl/fl^* mice to determine whether there is a more severe phenotype in the *Tbx1^Cre/+^ Kmt2d^fl/fl^* (conditional knockout [cKO]) mice. Conditional knockout of both copies of *Kmt2d* was confirmed using RT-qPCR, which showed significant depletion (*P* < 0.0001) of *Kmt2d* in the cKO compared with *Tbx1^Cre/+^* control embryos at E9.5 (Supplemental Figure 1E). Dissection of littermates at various stages during embryonic development showed expected Mendelian ratios for each genotype (Supplemental Figure 1F). Assessment at E16.5 did not reveal appreciable gross defects in either the cHet or the cKO embryos as compared with *Kmt2d^fl/fl^* controls (Figure 1A). We found that all cKO embryos survived embryogenesis (E17.5; Figure 1B, dashed line) but none survived past the first day after birth (P0; Figure 1B). We then systematically analyzed the phenotypes in resulting embryos, beginning with the craniofacial region, to determine the reason for lethality. Whole-body micro-CT scans were performed at E17.5 (Figure 1C), and craniofacial parameters were quantified. Differences in the head width/mandibular width ratio (Figure 1D), as well as the mandibular width/mandibular length ratio (Figure 1E), were slight but significant only between controls and cKO embryos. There was no statistical difference in these ratios between controls and cHet embryos, nor between cHet and cKO embryos, suggesting an intermediate phenotype that is subthreshold in the cHet embryos. The midface length/mandibular length ratio (Figure 1F) was significantly greater in cKO embryos compared with both controls and cHet embryos. Taken together, we found that the cKO embryos had slightly narrower heads with shorter but wider (posteriorly) mandibles as compared with controls. We observed normal skeletal structures (Figure 1C) except for the presence of an extra rib element at the C7 vertebra in most of the cKO embryos as compared with controls, suggesting partial disruption of the known homeotic role of KMT2D (27). Finally, microdissections and micro-CTs of control and cKO embryos at P0 showed normal organs on a gross level (Supplemental Figure 2, A and B), including a normal-appearing hyoid bone (Supplemental Figure 2C), which is known to be disrupted in *Tbx1* mutants (28).

Since these are not likely lethal defects on their own, we further assessed cause of lethality by examining litters at 6-hour intervals between P0 and P1. We found that 40% of newborns died within the first 6 hours after birth, and the rest died between 12 and 24 hours after birth (Figure 1G). To make sense of these 2 critical time points of lethality, we performed necropsy studies at the end of P0, which revealed an absence of milk in the stomach of the cKO newborns as compared with stage-matched controls (Figure 1H). This finding indicates a lethal suckling defect, consistent with the reported timing for neonatal murine death due to malnourishment (29). We did not identify an overt cleft palate (which occurs in *Tbx1*-null embryos), but we observed a submucosal/incomplete cleft, as evidenced by increased distance between the posterior rugae at the midline, which was seen in all mutants with varying severity (Figure 1I). Notably, palatal abnormalities are common in 22q11.2DS and include overt cleft as well as velopharyngeal insufficiency due to non-bony defects (30). Although of unique occurrence, we also noted one mutant with absence of multiple bones of the posterior palate (Supplemental Figure 2D). The observed mild craniofacial and palatal abnormalities together suggest impaired feeding and/or other causes of suckling impairment (29); however, they do not account for the smaller percentage of lethality that occurs in the first quarter of P0.

### Kmt2d conditional null mutant embryos have cardiac and aortic arch defects.

**Figure 2:**
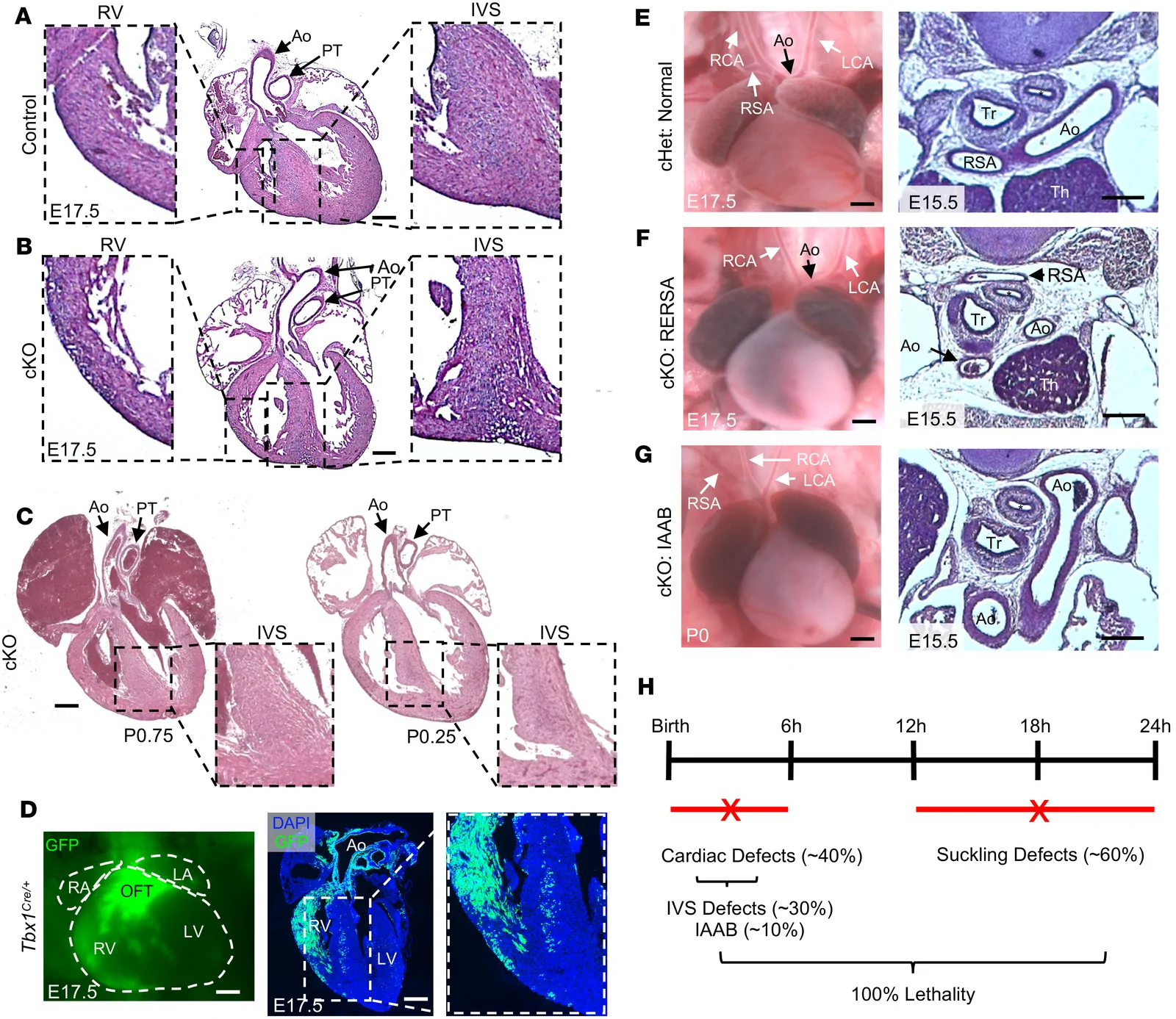
*Kmt2d*-cKO embryos have cardiac and aortic arch defects. (**A**) Control heart at E17.5 showing proper formation of the interventricular septum (IVS) and right ventricular (RV) myocardium (*n* = 3). (**B**) cKO littermate heart showing structural abnormalities in the IVS and RV myocardium (*n* = 10, 3 of which displayed this defect). (**C**) cKO hearts at P0.75 versus P0.25 showing difference in structure of the IVS (*n* = 2 per stage). (**D**) Left: Whole heart of E17.5 *Tbx1^Cre/+^* embryo with GFP reporter marking the lineage (*n* = 5). Right: GFP immunofluorescence on a section of an E17.5 *Tbx1^Cre/+^* heart (*n* = 3). (**E**) Gross view of an E17.5 heart and H&E-stained transverse section at E15.5 showing normal aortic branching in the cHet. (**F**) Gross view of an E17.5 heart and H&E-stained transverse section at E15.5 showing RERSA in the cKO embryos. (**G**) Gross view of a P0 heart and H&E-stained transverse section at E15.5 showing IAAB in the cKO. (**H**) Schematic of the different phenotypes observed in the *Kmt2d*-cKO embryos that are suggested to cause lethality at different time points during P0. Scale bars: 250 μm. *Esophagus; Ao, aorta; IAAB, interrupted aortic arch type B; IVS, interventricular septum; LA, left atrium; LCA, left common carotid artery; LV, left ventricle; OFT, outflow tract; PT, pulmonary trunk; RA, right atrium; RCA, right common carotid artery; RSA, right subclavian artery; RV, right ventricle; Th, thyroid; Tr, trachea.

Since congenital heart defects are a common cause of early neonatal lethality, and such defects are present in both 22q11.2DS and Kabuki syndrome, we performed gross and histological analysis of control (*Kmt2d^fl/fl^*) and cKO late-stage (>E15.5) embryos and P0 pups. Histology of hearts at E17.5 showed normal morphology in controls (Figure 2A), but myocardial hypoplasia in the interventricular septum (IVS) at the apex of the heart, as well as thinning of the adjacent right ventricular (RV) myocardium, in 30% of cKO mutants (Figure 2B). Assessment at E15.5 showed a similar percentage (30%) with disorganization in the same region of the IVS that resembled an incomplete muscular ventricular septal defect (VSD) (Supplemental Figure 3, A and B). Interestingly, although VSDs are common in both 22q11.2DS and Kabuki syndrome, we did not observe VSDs in the cKO mutant embryos. Assessment of P0 cKO littermate hearts supported this distinction in cardiac phenotype, showing an abnormally structured IVS in the P0.25 pup as compared with its P0.75 counterpart (Figure 2C), suggesting that the former may die earlier in P0 as a result of cardiac dysfunction. We therefore examined the *Tbx1* lineage in the late-stage heart by assessing the localization of GFP, which can be appreciated in the IVS and RV myocardium of the E17.5 heart grossly (Figure 2D). Anti-GFP immunofluorescence-stained cryosections at E17.5 (Figure 2D) and earlier at E15.5 (Supplemental Figure 3C) show that the *Tbx1* lineage extends into the right ventricle and part of the IVS (26, 31, 32). We found similar IVS/RV defects in a subset of *Tbx1*-null mutants (*Tbx1^Cre/fl^*; ~22%, *n* = 9) versus controls (*Tbx1^Cre/+^*) at E17.5 (Supplemental Figure 3, D–F), suggesting that loss of either *Tbx1* or *Kmt2d* could affect the development of the IVS at the apex of the heart.

Although *Tbx1*-null mice have fully penetrant PTA, we did not observe this phenotype or note other OFT abnormalities in cKO mutant embryos/neonates. We theorized that this could be due to functional redundancy between *Kmt2d* and another *Kmt2* family member such as *Kmt2c*, its paralog. To test this, we inactivated both genes using *Tbx1^Cre^* and found that the *Kmt2c*/*Kmt2d* double-cKO mice had similar perinatal lethality and phenotype to the *Kmt2d*-cKO embryos, displaying proper septation of the aorta and pulmonary trunk as compared with control littermates (Supplemental Figure 4, A and B). We performed this cross with 2 different constructs of a *Kmt2c*-floxed mouse, one with deletion in the SET domain (33) (data not shown) and one with deletion at exon 3 (34), with neither displaying a detectable increase in phenotypic severity. Further, we observed that mice with conditional inactivation of either construct of *Kmt2c* in the *Tbx1* lineage survived past weaning age, indicating disparate roles for *Kmt2c* and *Kmt2d* in this lineage despite their paralogous relationship (Supplemental Figure 4, C and D).

Since aortic arch branching anomalies are frequent in 22q11.2DS and *Tbx1*-mutant embryos, we assessed the aorta and its arterial branches. This was done by opening of the chest for gross assessment at E17.5 and by histological sectioning with hematoxylin and eosin (H&E) staining of embryos at E15.5 for confirmation of proper arterial branch points (Figure 2E). This revealed the presence of RERSA (Figure 2F) in 37% of cKO mutant embryos and IAAB (Figure 2G) in 11%. While RERSA is a non-lethal phenotype (35), IAAB is lethal in both humans and mice (36) and accounts for a subset of the early P0 lethality in cKO pups. Incidentally, we also noted that 31% of cHet embryos presented with RERSA as well, which was not found in the *Tbx1^Cre/+^* mice in the mixed genetic background, suggesting that inactivation of one copy of *Kmt2d* in the presence of *Tbx1* heterozygosity elicits low penetrance of this aortic branching phenotype. A summary of the proposed causes of lethality in the cKO is presented in Figure 2H.

### Kmt2d cKO embryos display increased penetrance of fourth PAA defects.

**Figure 3:**
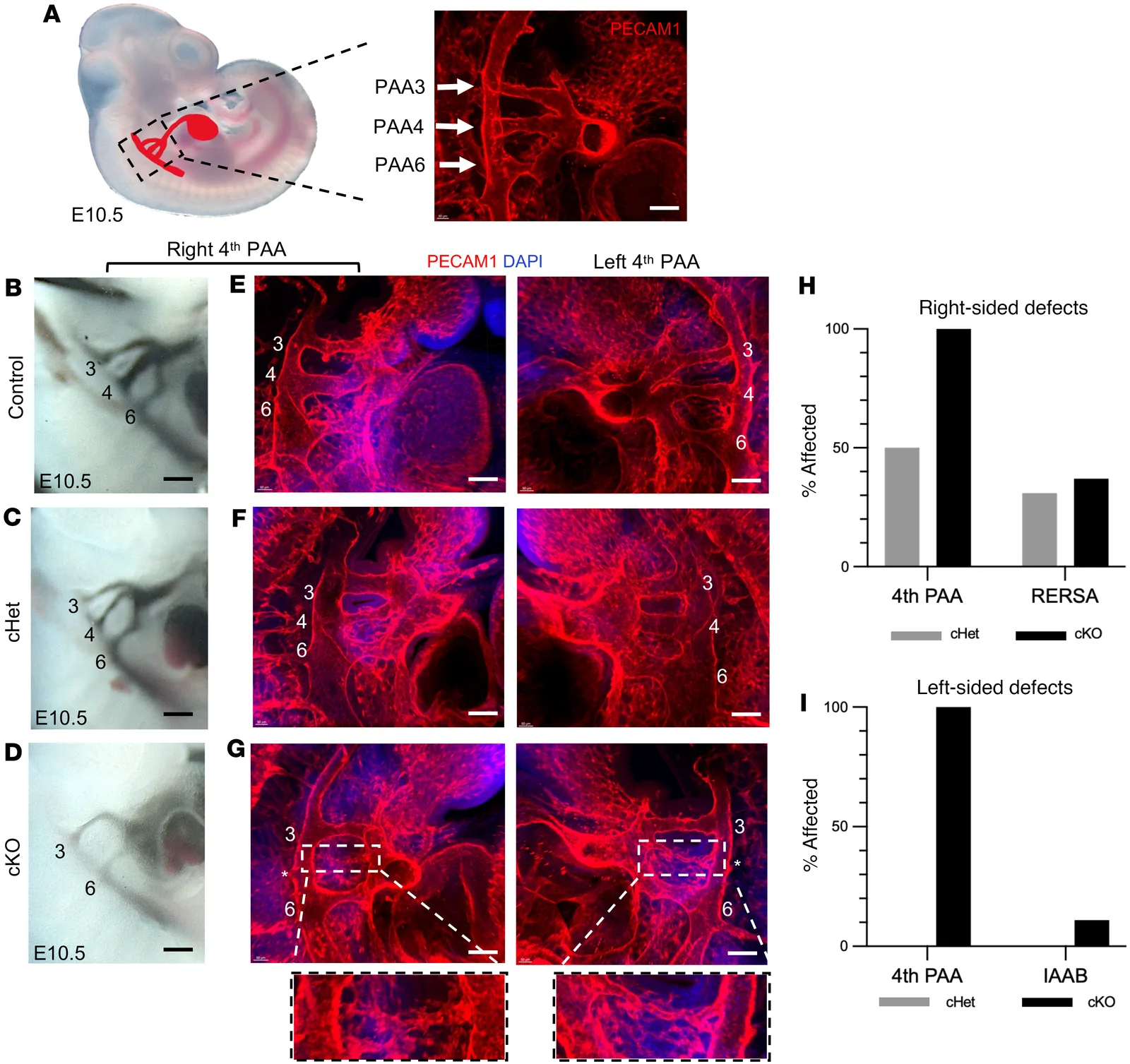
*Kmt2d*-cKO embryos have more severe fourth PAA defects than other genotypes. (**A**) E10.5 mouse embryo with graphical overlay of the dorsal aorta and PAAs 3–6 and a representative PECAM1 immunofluorescence staining of the third, fourth, and sixth PAAs of a control (*Kmt2d^fl/fl^*) embryo. (**B**–**D**) India ink injection of E10.5 right-sided PAAs in control, cHet, and cKO embryos shows that ink could not fill the fourth PAA in the cKO embryos, indicating absence of an artery lumen. (**E**–**G**) PECAM1 (red) and DAPI (blue) stain of right and left fourth PAAs in control, cHet, and cKO embryos at E10.5 showing that cKO embryos have an endothelial plexus (asterisks and insets) instead of a mature artery. (**H**) Summary of the right-sided defects in the cHet compared with the cKO embryos. RERSA occurred at reduced penetrance in both cHet and cKO embryos. (**I**) Summary of the left-sided defects in cHet compared with the cKO embryos. IAAB only occurred in the cKO mutant embryos and at reduced penetrance. Scale bars: (**A**) 100 μm, (**B**–**D**) 300 μm, (**E**–**G**) 100 μm. (**G**) Insets magnification, ×5.7. IAAB, interrupted aortic arch type B; PAA, pharyngeal arch artery; RERSA, retroesophageal right subclavian artery.

Because of the presence of both IAAB and RERSA in a subset of cKO pups, we looked earlier in developmental time at the fourth PAAs at E10.5. The left fourth PAA forms the portion of the aorta proximal to the left carotid artery, while the right fourth PAA forms the right subclavian artery (RSA); thus, defects in the former lead to IAAB and defects in the latter lead to RERSA (37). To do this, whole-mount immunofluorescence was performed on embryos at E10.5 using antibodies to PECAM1 to mark endothelial cells and visualize the third, fourth, and sixth PAAs (Figure 3A). India ink injection into the heart can identify whether the PAAs are patent, as is demonstrated in controls (*Kmt2d^fl/fl^*) and cHets at E10.5 that have proper filling of the third, fourth, and sixth PAAs (Figure 3, B and C). There was no filling of India ink in the fourth PAAs of cKO embryos (Figure 3D), indicating the absence of a fourth PAA lumen. Whole-mount PECAM1 immunofluorescence similarly shows proper formation of the PAAs in controls bilaterally (Figure 3E). Around half of the cHet embryos had proper fourth PAA formation bilaterally (Figure 3F), while the other half had hypoplasia or absence of the right fourth PAA. We observed a similar proportion of fourth PAA defects in the cHet and *Tbx1^Cre/+^* embryos, even though only the former displayed RERSA at late stage. Finally, 100% of cKO embryos had bilateral hypoplasia and/or absence of the fourth PAAs, which often presented as an endothelial plexus with no artery/lumen (Figure 3G). Despite the complete penetrance of bilateral fourth PAA defects in cKO embryos at E10.5, the corresponding late-stage defects, as noted above, only occurred in a fraction of cKO mutants. This discrepancy could be seen for both right- (Figure 3H) and left-sided (Figure 3I) defects, consistent with the partial recovery in fourth PAA defects that is widely reported in *Tbx1^+/–^* heterozygous mice (35). Information on the proportions of fourth PAA and aortic branching defects can be found in Table 1. Finally, using the *Mef2c-AHF-Cre* mice, we inactivated *Kmt2d* in the anterior second heart field (aSHF) lineage, from which the endothelium of the third, fourth, and sixth PAAs is derived (38). We found that there were no fourth PAA defects in this condition, indicating that the fourth PAA phenotype of the *Kmt2d* cKO in the *Tbx1* lineage is mediated by alteration of *Tbx1* function rather than direct contribution of *Kmt2d* to the fourth PAA endothelium (Supplemental Figure 5A). Further, we noted that the *Tbx1* lineage contribution to the fourth PAA was more limited compared with that of the aSHF lineage, suggesting that *Tbx1* may impact the fourth PAA endothelial cells in a non-autonomous manner as has been previously described (39) (Supplemental Figure 5, B–D).

### Kmt2d-cKO embryos exhibit thymus and parathyroid defects due to improper third pharyngeal pouch morphogenesis.

**Figure 4:**
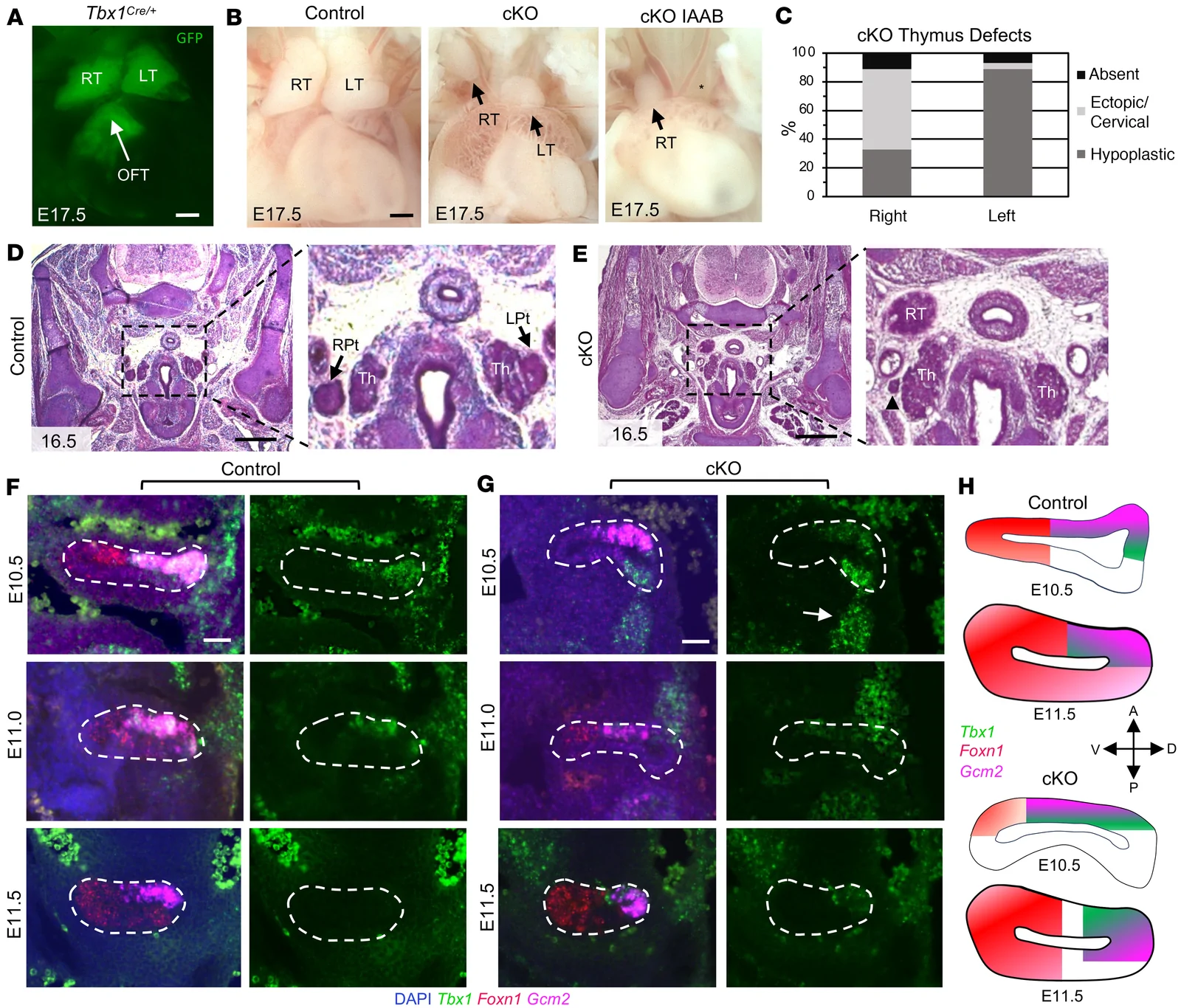
*Kmt2d*-cKO embryos exhibit thymus and parathyroid gland defects and third pharyngeal pouch differentiation defects. (**A**) Whole-mount view of the thymus anterior to the heart in a *Tbx1^Cre/+^* embryo at E17.5 using GFP to mark the *Tbx1* lineage (*n* = 5). (**B**) Whole-mount views of the thymus and heart in the different genotypes of interest. Control (*Kmt2d^fl/fl^*) embryos at E17.5 have intact thymus lobes bilaterally, while the cKO embryos present with variable defects. The majority of cKO embryos presented with left and right lobe hypoplasia and ectopia (cKO), but several presented with right lobe hypoplasia and absence (indicated by asterisk) of the left lobe (cKO, IAAB). (**C**) Graph depicting the difference in severity of left- versus right-sided thymus defects in cKO embryos. (**D**) Transverse H&E section of a control embryo at E16.5 showing proper formation of the parathyroid glands. (**E**) Transverse H&E section of a cKO embryo at E16.5 showing absence of the left parathyroid gland and a hypoplastic right parathyroid gland (arrowhead). (**F**) RNAscope analysis of paraffin tissue sections in the region of the third pharyngeal pouch using probes for *Tbx1*, *Foxn1*, and *Gcm2* in controls shows normal gene expression patterns from E10.5 to E11.5 (E10.5, *n* = 5; E11/11.5, *n* = 3). (**G**) RNAscope analysis of the third pharyngeal pouch in cKO embryos shows differences in pouch formation and reduced expression of *Foxn1* and *Gcm2* that can be appreciated from E10.5 to E11.5 (E10.5, *n* = 5; E11/11.5, *n* = 3). (**H**) Illustration of the expression patterns seen in the third pharyngeal pouch of control versus cKO embryos at E10.5 and E11.5. Scale bars: (**A** and **B**) 250 μm, (**D**–**G**) 50 μm. A, anterior; D, dorsal; LPt, left parathyroid gland; LT, left thymus lobe; OFT, outflow tract; P, posterior; RPt, right parathyroid gland; RT, right thymus lobe; Th, thyroid gland; V, ventral.

Another obvious defect in cKO mutant embryos upon opening of the chest cavity was the presence of thymus gland defects, which is a common occurrence in 22q11.2DS and *Tbx1*-mutant mouse models (17, 18, 40). GFP expression in *Tbx1^Cre/+^* at E17.5 showed the presence of the *Tbx1* lineage in the thymic lobes, presumably the thymic epithelial cells derived from the pharyngeal endoderm lineage (41) (Figure 4A). Controls (*Kmt2d^fl/fl^*) showed normal thymus lobes bilaterally, while cKO embryos displayed a variety of defects ranging from hypoplasia to ectopia to complete absence of the thymus (Figure 4B). It is interesting to note that right-sided thymus defects were more severe than left-sided defects (Figure 4C) and that the most severe left-sided defects occurred in conjunction with IAAB (as seen in Figure 4B). We then assessed for parathyroid gland defects as well, since both the thymus and parathyroid glands arise from the third pharyngeal pouch (42). Controls had proper localization of the parathyroid glands (Figure 4D), but assessment of the cKO embryos revealed bilateral parathyroid gland defects (Figure 4E), which was also observed in cHets (Table 2). Our laboratory previously reported that 38% of *Tbx1* heterozygotes of mixed background had parathyroid defects (24), thus indicating that there is an increased frequency of parathyroid gland defects in the cHet and cKO embryos described here. The parathyroid glands are small at this stage, and we therefore did not subclassify defects, as they likely include a combination of hypoplasia (Figure 4E, arrowhead), ectopia, and/or absence. These defects are summarized in Table 2. To understand the origin of the thymus and parathyroid abnormalities seen in the cKO mutant embryos, we performed RNAscope in situ hybridization on embryo tissue sections of control and cKO embryos at E10.5 and E11.5. We investigated the third pharyngeal pouches using markers for the primitive parathyroid (*Gcm2*) and thymus (*Foxn1*) glands. Proper patterning involves anterior-dorsal expression of *Gcm2* and ventral expression of *Foxn1* at E10.5, followed by an increase of *Foxn1* expression into the posterior-ventral region of the third endodermal pouch until its expression pattern fully complements that of *Gcm2* by E11.5 (Figure 4F). The expression of *Tbx1* overlapped with *Gcm2* at both E10.5 and E11.5 (Figure 4F). In the cKO embryos, expression of both *Gcm2* and *Foxn1* was reduced (Figure 4G), while the pattern of *Tbx1* matched *Gcm2* but also showed some possible ectopic expression (Figure 4G, white arrow). Aside from abnormal expression patterning, the structure of the pharyngeal pouch itself was malformed, particularly at E10.5, as is graphically represented in Figure 4H. One cKO embryo showed a particularly small third pharyngeal pouch at E11.5 (Supplemental Figure 6, A and B), which we posit would have led to a late-stage phenotype on the more severe end of the spectrum of defects; this phenotype has also been reported in the *Ezh2* cKO in the *Tbx1* lineage (9). Thus, there was variability in the reduced expression and structural dysmorphism of the third pharyngeal pouch, consistent with the variability seen in the defects of the later-stage counterparts of the thymus and parathyroid glands.

### Kmt2d-cKO embryos have developmental delay of the fourth pharyngeal arch.

**Figure 5:**
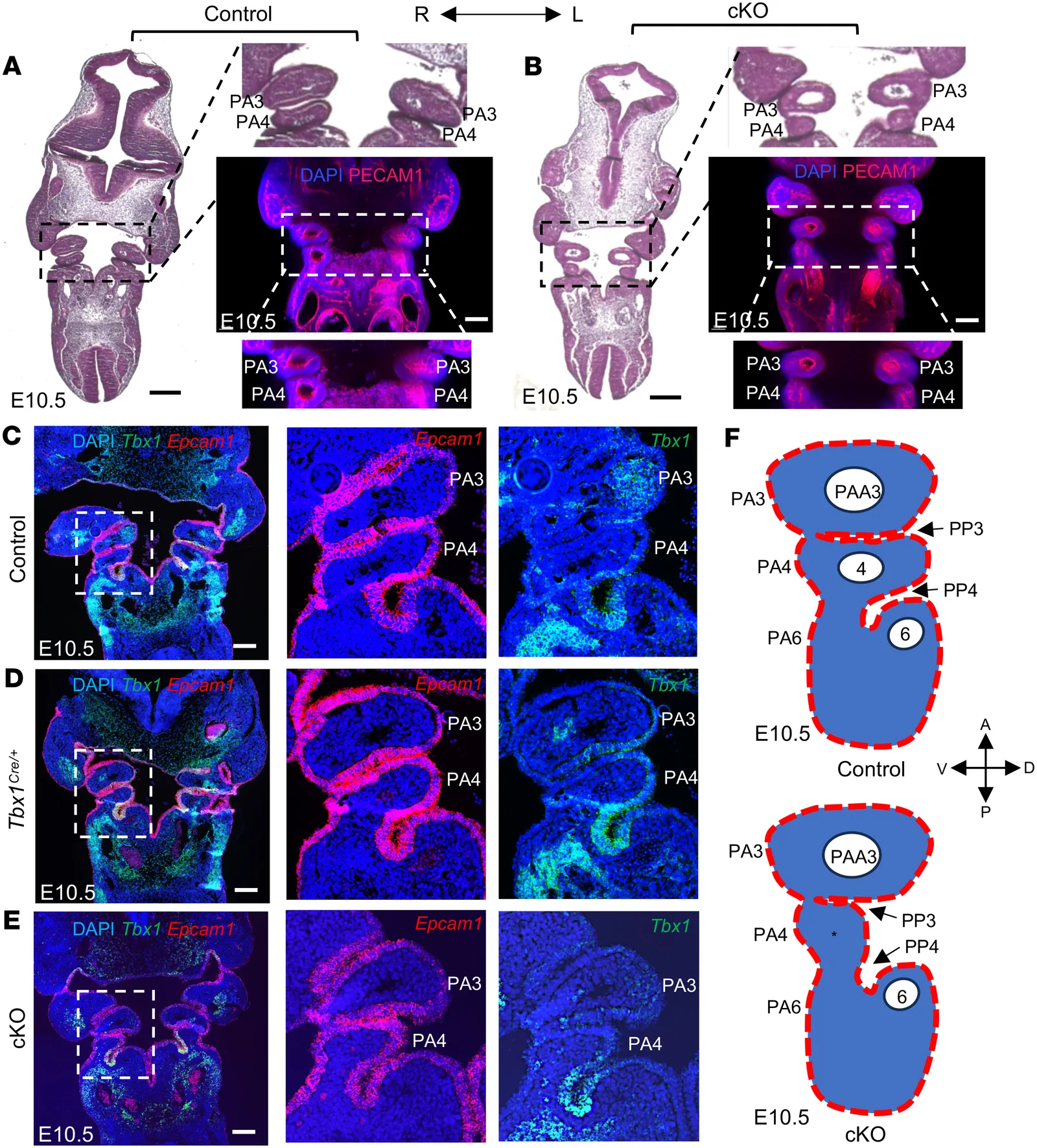
*Kmt2d*-cKO embryos have abnormal development of the third and fourth pharyngeal arches. (**A**) Frontal images of control (*Kmt2d^fl/fl^*) embryos at E10.5 showing proper formation of the third and fourth pharyngeal arches (PA) using H&E staining (*n* = 3) and whole-mount immunofluorescence with PECAM1 and DAPI (*n* = 5). (**B**) Frontal images of cKO embryos at E10.5 showing hypoplasia of the fourth PA using H&E staining (*n* = 3) and whole-mount immunofluorescence with PECAM1 and DAPI (*n* = 5). (**C**) Frontal RNAscope analysis of DAPI, *Tbx1*, and *Epcam1* on a control embryo at E10.5 with single-channel magnified views of *Epcam* and *Tbx1* (*n* = 5). (**D**) Frontal RNAscope of DAPI, *Tbx1*, and *Epcam1* on a *Tbx1^Cre/+^* embryo at E10.5 with single-channel magnified views of *Epcam* and *Tbx1* (*n* = 4). (**E**) Frontal RNAscope of DAPI, *Tbx1*, and *Epcam1* on a cKO embryo at E10.5 with single-channel magnified views of *Epcam* and *Tbx1* (*n* = 5). (**F**) Illustration of the structural differences in the pharyngeal arches between control and cKO embryos. Scale bars: 250 μm. PA, pharyngeal arch; PP, pharyngeal pouch.

Because of the abnormalities in the third pharyngeal pouch, we next examined the overall morphology of the pharyngeal apparatus for defects using H&E histological analysis at E10.5. At this stage, the third, fourth, and sixth pharyngeal arches have formed, although the sixth arch is small and difficult to detect even in normal embryos. The controls (*Kmt2d^fl/fl^*) had proper formation of the pharyngeal arches, which was visualized with frontal H&E-stained sections as well as with whole-mount PECAM1 and DAPI staining that shows the proper position of the third and fourth PAA lumens relative to their respective arches (Figure 5A). In cKO embryos these assessments revealed notable hypoplasia of fourth pharyngeal arch and clear absence of a fourth PAA lumen (Figure 5B). Sagittal view of DAPI-stained embryos at E10.5 similarly shows a decrease in third pharyngeal endoderm pouch size, as well as diminished distance between the pouches due to the absence of the fourth PAA (Supplemental Figure 6, C and D). Because of these structural differences, we wanted to assess the formation of the cranial nerves that correspond to each of the pharyngeal arches by performing whole-mount immunofluorescence using an antibody against β_III_-tubulin. However, while some of the cKO embryos showed an increased incidence of fusion between cranial nerves IX and X compared with controls and cHets (Supplemental Figure 6, E and F), this variation was not fully penetrant and appeared in some of the controls as well (Supplemental Figure 6G). Thus, despite structural changes to the caudal pharyngeal apparatus at E10.5, there is limited impact on early cranial nerve patterning mediated by *Kmt2d* in the *Tbx1* lineage. To clarify the nature of the abnormal pharyngeal morphology, we performed RNAscope on frontal E10.5 sections with probes for *Epcam* and *Tbx1* to assess for proper pharyngeal epithelium morphogenesis and *Tbx1* expression patterning, respectively. In addition to our *Kmt2d^fl/fl^* control (Figure 5C), we also used a *Tbx1^Cre/+^* control (Figure 5D) to compare with cKO embryos (Figure 5E), as *Tbx1* heterozygosity alone has been shown to cause pharyngeal apparatus structural changes with fourth PAA hypoplasia (43). The results showed *Epcam* expression marking the pharyngeal endoderm and ectoderm, with proper invagination of the third and fourth pouches, as well as comparable *Tbx1* expression, among the 3 conditions. The DAPI staining, however, showed that there was hypoplasia of the cells within the fourth arch of the cKO embryos, consequently limiting the area occupied by the third pouch as compared with both the *flox* and *Cre* controls (illustration in Figure 5F). We therefore wanted to assess earlier expression of *Tbx1* at E9.5, which is when the third pharyngeal arch has developed and the fourth is forming (44). Using whole-mount in situ experiments, we found that, compared with controls (Supplemental Figure 7, A and B), *Tbx1* expression and/or the pharyngeal pouches appeared abnormal at the midline of the boundary between the third and fourth arches on both sides of the embryo (Supplemental Figure 7, C and D). Digital frontal sections were reconstructed from these images showing proper invagination of the third pharyngeal pouch (Supplemental Figure 7E) and prominent *Tbx1* expression in the forming fourth pouch (Supplemental Figure 7F) in the control. The overall structure of the pharyngeal apparatus showed proper morphology when visualized with DAPI alone (Supplemental Figure 7G). In comparison, cKO embryos had delayed and/or incomplete formation of the third (Supplemental Figure 7H) and fourth (Supplemental Figure 7I) pharyngeal pouches, and fourth arch hypoplasia, which can be visualized with DAPI alone (Supplemental Figure 7J). While the expression of *Tbx1* in the fourth pouch of cKO embryos at E9.5 did appear reduced, this is likely due to *Tbx1* heterozygosity rather than the effects of *Kmt2d* inactivation; this is supported by qRT-PCR at E9.5 showing no significant difference between *Tbx1* expression levels of *Tbx1^Cre/+^* controls, cHets, and cKOs (Supplemental Figure 7K).

### Single-cell transcriptomics reveals mild downregulation of genes with broad biological functions.

**Figure 6:**
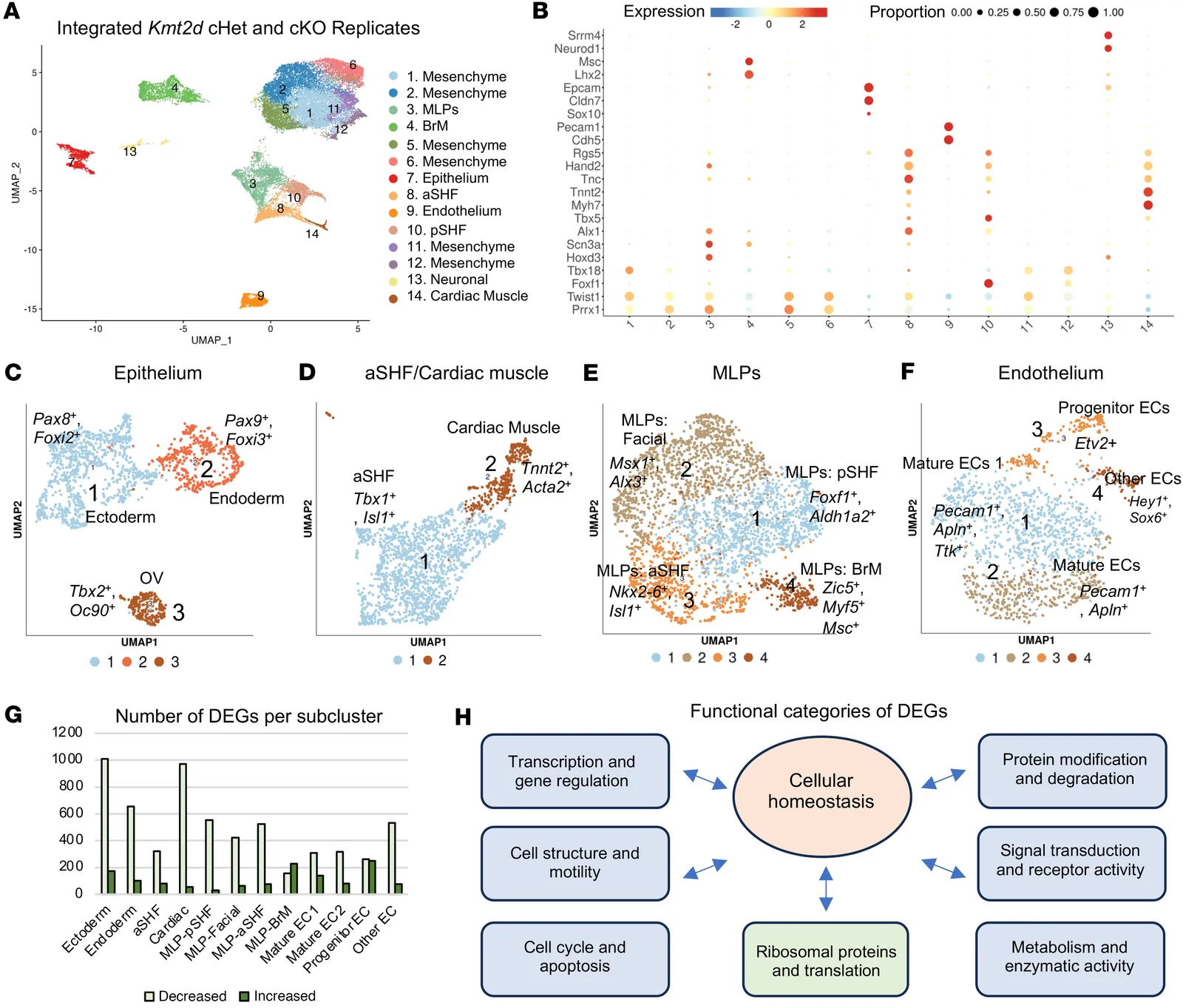
Single-cell RNA sequencing of cHet versus cKO embryos at E9.5 identifies differentially expressed genes in specific populations within the embryonic pharyngeal apparatus and heart. (**A**) Representative integrated UMAP of the 2 replicates of the scRNA-seq performed with cHet and cKO samples at E9.5. Clusters are labeled by color and were identified using known cell type markers. (**B**) Dot plot of the marker genes used to label the integrated cluster cell types. (**C**–**F**) Integrated UMAP plots of subcluster analysis of the epithelium (**C**), aSHF/cardiac muscle (**D**), MLPs (**E**), and endothelium (**F**). (**G**) Graph depicting the number of differentially expressed genes (DEGs) that were increased or decreased in expression within each subcluster. (**H**) Illustration of the results showing that DEGs of all cell subcluster populations (**C**–**F**) comprise varied biological processes, both increased and decreased in cKO embryos. aSHF, anterior second heart field; BrM, branchiomeric muscle; ECs, endothelial cells; MLPs, multilineage primed progenitors; OV, otic vesicle; pSHF, posterior second heart field.

To explain the molecular mechanism between the *Kmt2d* cHet versus cKO phenotype in the *Tbx1* lineage, we used single-cell RNA sequencing (scRNA-seq) to evaluate gene expression level changes. We performed the experiment at E9.5, because this is when *Tbx1* expression is at its highest (21). The pharyngeal apparatus and heart from E9.5 embryos were microdissected, and *Tbx1*-lineage cells were purified using fluorescence-activated cell sorting (Supplemental Figure 8, A–C). We computationally integrated and clustered 2 replicates of cHet and cKO datasets (Supplemental Figure 8D) and identified 14 cell types using marker genes characterized in a previous report (21) (Figure 6, A and B, and Supplemental Figure 9, A–D). To calculate differentially expressed genes (DEGs) between cHet and cKO samples, we used the MAST (Model-based Analysis of Single-cell Transcriptomics) approach, a statistical framework that accounts for dropout and expression variation in scRNA-seq datasets (45). This revealed that the majority of the DEGs had low fold changes and that they function in a wide array of basic biological processes.

**Figure 7:**
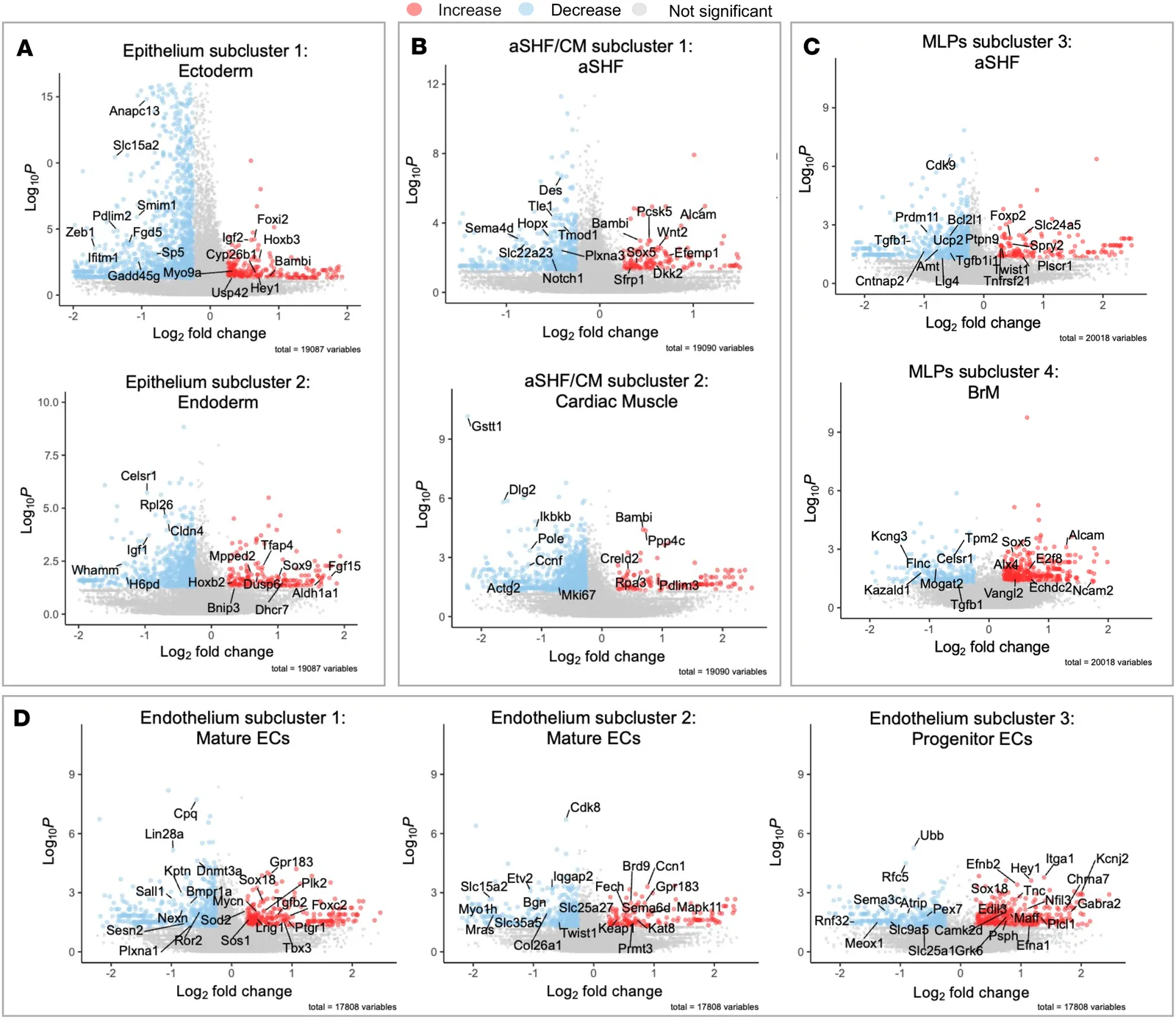
Volcano plots from scRNA-seq show gene expression changes in cHet versus cKO embryos at E9.5. (**A**) Volcano plots of the DEGs in the ectoderm and endoderm subclusters of the epithelium. (**B**) Volcano plots of the DEGs in the aSHF with cardiac muscle (aSHF/CM) subclusters. (**C**) Volcano plots of the aSHF and BrM subclusters of the MLPs. (**D**) Volcano plots of the DEGs in the mature (1 and 2) and progenitor EC subclusters of the endothelium. aSHF, anterior second heart field; BrM, branchiomeric muscle; ECs, endothelial cells; MLPs, multilineage primed progenitors.

To increase the resolution of the analysis, we performed subcluster analysis on some of the major cell types of the caudal pharyngeal arches, including the epithelium (Figure 6C), aSHF/cardiac muscle (Figure 6D), multilineage primed progenitors (MLPs) (Figure 6E), and the endothelium (Figure 6F). Most of the DEGs were decreased in expression in the 4 cell types assessed, a trend that was present across subclusters (Figure 6G). To determine the biological functions of these genes, analysis of all subclusters in the 4 selected cell types was performed, and these results are summarized in Figure 6H. Overall, we found both downregulated and upregulated DEGs whose gene products are involved in basic aspects of cellular homeostasis, including gene expression, signaling, metabolism, cell cycle regulation, protein modification, and ribosomal translation. Volcano plots were generated from the single-cell data to visualize the relative fold changes of DEGs and to highlight genes of interest (Figure 7). A full list of DEGs for each subcluster can be found in Supplemental Table 1. The epithelium (cluster 7) includes the pharyngeal pouch endoderm (subcluster 1), cleft ectoderm (subcluster 2), and otic vesicle (subcluster 3) as identified by cell type–specific marker genes (Figure 7A). The aSHF and cardiac muscle subclusters (clusters 8 and 14, respectively) are important contributors to the developing heart (Figure 7B). The MLPs (cluster 3) contribute to structures derived from the caudal pharyngeal arches, and we subdivided this cluster into posterior second heart field (pSHF; subcluster 1), facial (subcluster 2), aSHF (subcluster 3), and branchiomeric muscle (subcluster 4) cell types (Figure 7C and Supplemental Figure 9E). The endothelium contributes to the PAAs, so to assess underlying transcriptomic changes we subdivided it into mature (subclusters 1 and 2), progenitor (subcluster 3), and other (subcluster 4) endothelial cell types (Figure 7D and Supplemental Figure 9F). The predominance of mildly but significantly downregulated DEGs suggests that the loss of *Kmt2d* in the *Tbx1* lineage leads to a subtle but broad shift in expression of genes constituting essential functional categories. Interestingly, neither *Tbx1* nor *Kmt2d* had noteworthy changes in expression when the different clusters/cell types were compared across conditions (Supplemental Figure 10, A and B), suggesting that their relationship is cooperative rather than hierarchical.

### Kmt2d and Tbx1 have shared and unique roles in the Tbx1 lineage.

**Figure 8:**
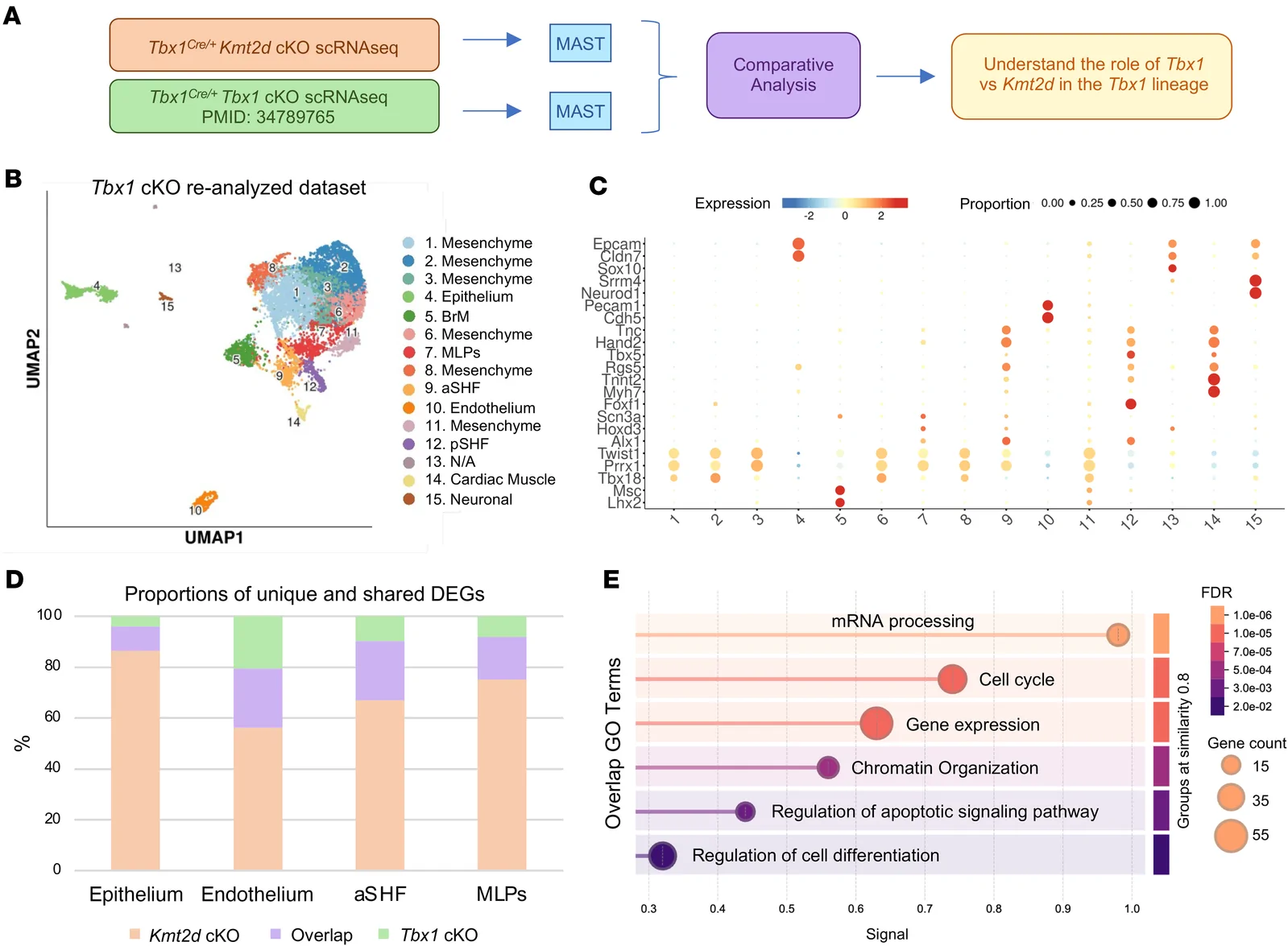
Comparison of the transcriptome from *Kmt2d* and *Tbx1* datasets at E9.5. (**A**) Schematic of the method for comparing the DEGs in *Kmt2d*-cKO and *Tbx1*-cKO embryos. The MAST method was used to reanalyze *Tbx1^Cre^* versus *Tbx1^Cre/fl^* (*Tbx1*-cKO) scRNA-seq data from E9.5 embryos and to compare the resulting DEGs with the ones found in the dataset of the second replicate of the *Kmt2d* cKO (Rep2). (**B**) UMAP of the reanalysis of data from *Tbx1*-cKO embryos at E9.5 with cell cluster identification using the same marker genes as described above. (**C**) Dot plot of the marker genes used to label the integrated cluster cell types. (**D**) Graph of the different proportions of downregulated DEGs that are shared and unique to the different mutant embryos (*Kmt2d* cKO vs. *Tbx1* cKO) in cell clusters that may contribute to observed phenotypes. (**E**) GO terms identified from the downregulated DEGs (*n* = 253) shared/overlapping between the epithelium, endothelium, aSHF, and MLPs of both datasets. aSHF, anterior second heart field; BrM, branchiomeric muscle; MLPs, multilineage primed progenitors; pSHF, posterior second heart field.

**Figure 9:**
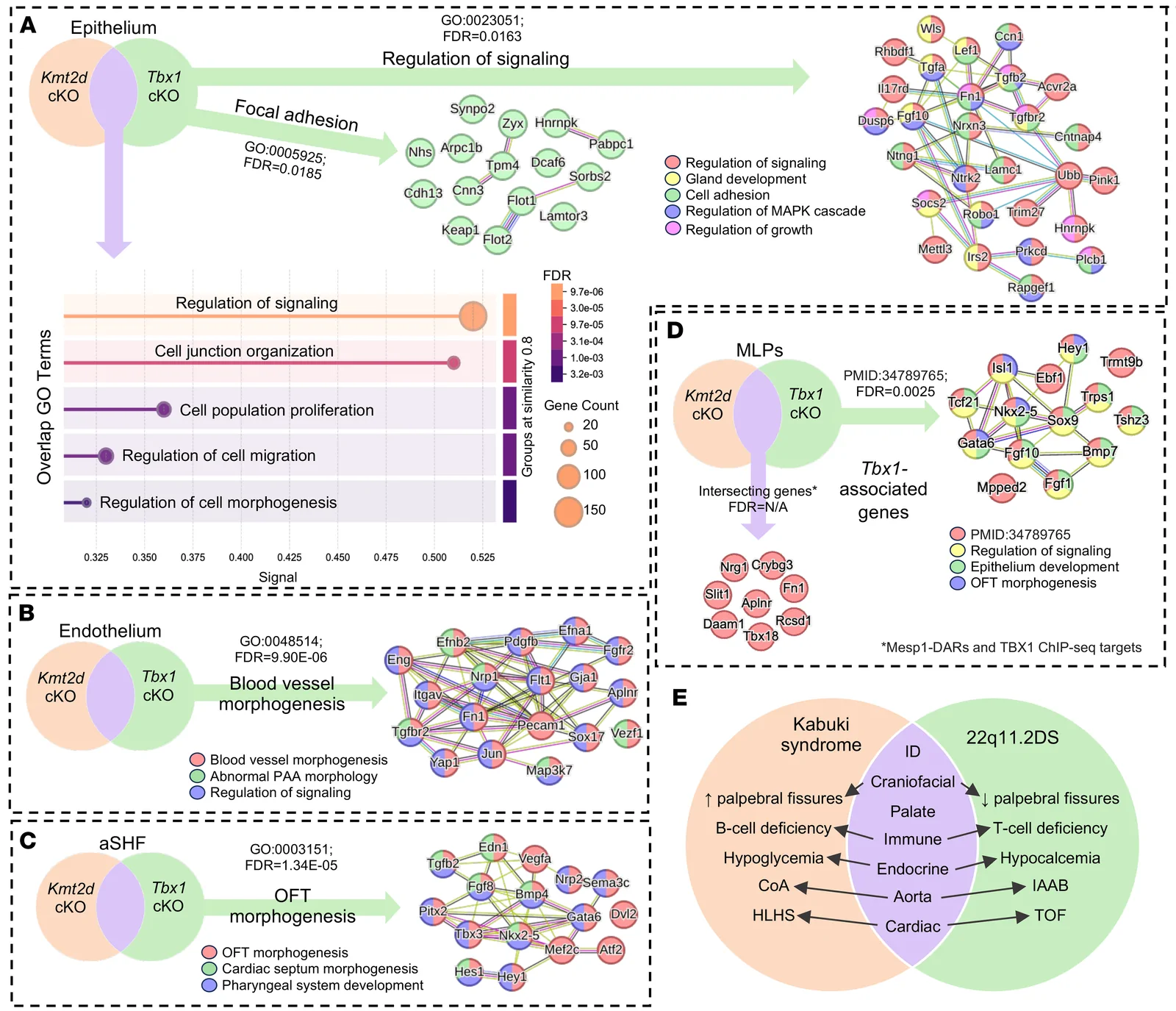
*Kmt2d* and *Tbx1* have shared and separate functions in the *Tbx1* lineage at E9.5. The orange circles in each panel represent DEGs in *Kmt2d*-cKO embryos, whereas the green circles represent DEGs in *Tbx1*-cKO embryos. The intersection of the two circles is in purple and represents shared DEGs. The color of the arrows indicates the source of the highlighted DEGs. (**A**) Comparative analysis of the epithelial cell clusters reveals shared GO terms related to cell proliferation and migration. Gene sets unique to the *Tbx1*-cKO embryos include focal adhesion and regulation of signaling. (**B**) The overlap of DEGs in the *Tbx1*- and *Kmt2d*-cKO endothelium reveals no shared DEGs relevant to endothelial cell function. The *Tbx1*-cKO data had unique DEGs enriched for blood cell morphogenesis. (**C**) Comparative analysis of the aSHF data shows a subset of DEGs related to cardiac OFT morphogenesis that are unique to the *Tbx1*-cKO dataset. (**D**) Comparison of the DEGs found in the MLPs reveals a significant proportion of known *Tbx1*-associated genes that are unique to the *Tbx1*-cKO data. However, manual assessment of the overlapped genes between the two datasets revealed *Tbx1*-associated genes that are also known to intersect with other relevant datasets. (**E**) Venn diagram showing a comparison of the phenotypes between Kabuki syndrome and 22q11.2DS. There is large overlap in the impacted systems, but the classical clinical presentation for each syndrome is distinct. aSHF, anterior second heart field; DARs, differentially accessible regions; MLPs, multilineage primed progenitors; OFT, outflow tract; PAA, pharyngeal arch artery.

To ascertain whether *Kmt2d* can serve as a modifier of *Tbx1*, we reanalyzed scRNA-seq data from a study of *Tbx1^Cre/+^* versus *Tbx1^Cre/fl^* (*Tbx1*-cKO) embryos in the same genetic background at E9.5 (21). These data consisted of one replicate each of pooled control and mutant embryos that we compared with our second replicate (Rep2) dataset as it had deeper sequencing and better overall cell quality. If *Kmt2d* modifies the *Tbx1*-cKO mutant phenotype, then we should observe some shared genes relevant to the general biological functions outlined above (Figure 7), and unique genes for the more severe phenotypes observed only in the *Tbx1-*cKO mutant embryos. We used the same pipeline and statistical methods to compare the MAST-derived DEGs from both datasets (Figure 8A). The cell clusters for the *Tbx1*-cKO dataset were identified using the same gene markers as those used in both *Kmt2d*-cKO datasets (Figure 8, B and C). We focused on the clusters consisting of epithelium, endothelium, aSHF, and MLPs to compare the shared and unique DEGs, as these were compelling based on our analysis and the known *Tbx1* literature. The *Kmt2d-*cKO dataset had a much larger number of DEGs but with overall smaller fold changes compared with the *Tbx1-*cKO data; however, many of the DEGs overlapped between the 2 datasets and between clusters (Figure 8D). We therefore analyzed the overlapping *Tbx1*-cKO/*Kmt2d*-cKO DEGs that were identified in all 4 aforementioned clusters and found Gene Ontology (GO) terms largely related to transcription, cell cycle, and other homeostatic pathways (Figure 8E), thus mirroring the pattern of DEGs identified in the *Kmt2d*-cKO individual analysis. In the epithelium, we found that the DEGs that overlapped between the datasets were largely related to signaling, cell morphogenesis/proliferation, and cell junction (Figure 9A). The DEGs unique to the *Tbx1*-cKO epithelium (i.e., that were not also found in the *Kmt2d* cKO) showed differences that included GO functions related to regulation of signaling and focal adhesion. In the endothelium, we did not find any shared terms that were relevant to endothelial cells, but the *Tbx1*-cKO data were enriched for DEGs involved in blood vessel morphogenesis (Figure 9B). Similarly, the aSHF analysis revealed unique *Tbx1*-cKO DEGs relevant to OFT morphogenesis not found in the *Kmt2d*-cKO data (Figure 9C). Finally, analysis of the MLPs revealed a significant subset of DEGs unique to the *Tbx1*-cKO dataset that were identified in the original *Tbx1*-cKO study, confirming that there were substantial changes to gene expression in *Tbx1*-cKO embryos that were not present in the *Kmt2d*-cKO embryos (Figure 9D). This is expected given the discernibly less severe phenotype present in *Kmt2d*-cKO embryos compared with the *Tbx1*-cKO embryos. However, we then manually examined the overlapping DEGs in the MLPs for genes that are relevant to *Tbx1* function. To do this, we compared them with a previously published list of 21 genes known to be heavily implicated in *Tbx1* function as they have been identified across multiple datasets, including *Tbx1*-cKO MLP DEGs, *Mesp1^Cre^*-mediated *Tbx1*-dependent differentially accessible regions, and TBX1 ChIP-seq targets (21). Of the 21 intersected genes, 8 were present in the overlapped MLPs described here, indicating a possible intertwined role of *Kmt2d* with *Tbx1* in these specific genetic pathways (Figure 9D). This is consistent with the clinical parallels between patients with 22q11DS or Kabuki syndrome, which have both shared and distinct phenotypes (Figure 9E). Thus, we confirm that there are some shared general biological processes between the *Kmt2d*-cKO and *Tbx1-*cKO datasets that may explain the subtle but remarkable ways in which *Kmt2d* modifies the phenotype of *Tbx1* haploinsufficiency.

## Discussion

When considering the syndromic presentation of 22q11.2DS and Kabuki syndrome, the overall phenotypic similarities in patients are evident, with overlap in many systems including, but not limited to craniofacial/skeletal, immune, endocrine, and cardiac. Despite this, the stereotypical manifestations differ between syndromes. Recent genomic analysis of patients with 22q11.2DS identified *KMT2D* among a list of chromatin modifier genes that may interact with *TBX1* to modulate cardiac phenotypic severity (5). Further, when we revisited the clinical manifestations of the 2 patients with *KMT2D* variants from this 22q11.2DS cohort, we found that, aside from CTDs, both presented with thymic atrophy/aplasia and velopharyngeal anomalies. These are phenotypes observed in 22q11.2DS, Kabuki syndrome, and the *Kmt2d*-cKO model described here, further illustrating that *TBX1* and *KMT2D* function within overlapping developmental programs. In this study we found evidence that *Kmt2d* is essential in the *Tbx1* lineage of the pharyngeal apparatus, such that its absence in this lineage during development leads to a spectrum of phenotypes that resemble mouse models of 22q11.2DS. The observed defects in cKO embryos, particularly of the thymus/parathyroid glands and aortic arch, result from a developmental delay of the caudal pharyngeal apparatus. Single-cell transcriptomic analysis demonstrates that this is likely due to the convergent roles in downstream gene regulation of shared biological processes, particularly affecting the pharyngeal epithelia, SHF mesoderm, and MLPs. Overall, our data, along with previous human genetic studies (5), show that *Kmt2d* serves as a modifier gene of select *Tbx1* functions in pharyngeal phenotypes.

Both *Kmt2d-*cKO and *Tbx1*-null (or *Tbx1^Cre/fl^*) embryos die perinatally, indicating essential developmental impacts on survival. The *Tbx1*-null embryos have phenotypes that readily explain perinatal lethality, including an overt cleft palate, absence of the hyoid bone, and a PTA with or without VSD (46). The definitive cause of lethality in *Kmt2d*-cKO neonates, however, was more difficult to establish, as the presenting phenotypes are fewer and milder. Assessment of cKO micro-CTs at E17.5 ruled out the aforementioned defects found in *Tbx1* nulls but revealed mild craniofacial differences of the face, palate, and mandible. Interestingly, similar and more severe phenotypes have been observed in *Tbx1* and *Kmt2d* mouse models, as well as in patients with 22q11.2DS and Kabuki syndrome (1, 47–49). The absence of milk in the stomachs of the cKO neonates at the end of P0, along with mild defects of the secondary palate, suggest a suckling defect and consequent lethality due to malnutrition. Feeding difficulties could also result from pharyngeal muscle hypotonia and/or cranial nerve IX and X defects, which occur in *Tbx1*-null embryos (44, 50). It is therefore possible that a combination of mild craniofacial and cranial nerve irregularities contributes to the perinatal lethality in cKO embryos, although our assessment of the cranial nerves at E10.5 was unremarkable. In addition to craniofacial abnormalities, we identified a subset of cKO pups that die early in P0 as a result of IAAB and possible cardiac IVS defects, the latter of which have been previously exhibited in some *Kmt2d* mouse models (25).

Global loss of *Kmt2d* results in early embryonic lethality, conveying the ubiquitous role that it plays in development and the need for conditional inactivation to study its specific functions (25). *Kmt2d* inactivation using *Mef2c-AHF-Cre* has been shown to result in mid-gestational lethality with a PTA and IVS defects (25). We therefore expected that loss of *Kmt2d* in the *Tbx1* lineage would result in a PTA, as the *Tbx1^Cre^* and *Mef2c-AHF-Cre* lineages overlap in the SHF and *Tbx1*-null mutants have a fully penetrant PTA (44, 51). Possible functional redundancy was addressed by conditional inactivation of both *Kmt2d* and its paralog *Kmt2c*, but OFT defects were still not observed. A subset of our *Kmt2d*-cKO embryos, however, did have structural defects of the RV and IVS myocardium, a phenotype that we then observed with reduced penetrance in *Tbx1*-null embryos. These intracardiac defects, particularly disorganization of the IVS, are reminiscent of the *Mef2c-AHF-Cre* *Kmt2d*-cKO phenotype (25). Thus, our data support function of *Kmt2d* within these cells and reveal a previously reported but understudied role of the contribution of the *Tbx1* lineage to the RV and IVS myocardium in the apex of the heart (26, 31, 32). Mild myocardial hypoplasia has been shown to cause perinatal lethality (52), possibly explaining how these cardiac defects, in addition to IAAB, can cause a subset of the lethality that occurs early in P0. We suggest that the relatively mild cardiac phenotype in cKO embryos using *Tbx1^Cre^* is due to the timing and cell type differences of the *Tbx1^Cre^* expression domain and lineage.

The aortic branching phenotypes that we observed are characteristic of those seen in *Tbx1* mutants as they originate from fourth PAA defects early in development. In fact, in studies testing for compound heterozygosity of *Tbx1* with other genes, increased penetrance of fourth PAA defects is often used as an indicator of a genetic interaction in double-heterozygous embryos (8, 53–55). Thus, the delicate balance in *Tbx1* heterozygosity, which results in partially penetrant fourth PAA defects and subsequent aortic arch anomalies (17), is further compromised by insult to additional genes that act downstream or in the same pathway. We observed this in the *Kmt2d*-cHet embryos, which, despite not having an increased frequency of fourth PAA defects compared with *Tbx1* heterozygotes, displayed low penetrance of RERSA not seen in the *Tbx1^Cre/+^* embryos in a mixed genetic background. Further, we observed that 100% of cKO embryos had fourth PAA defects, while late-stage embryos displayed RERSA and IAAB at reduced penetrance. As with other mouse models, there is recovery of fourth PAA defects during embryogenesis (17, 35, 56), but it can be observed here that reduced dosage of *Kmt2d* attenuates this mechanism. The absence of fourth PAA defects when *Kmt2d* is conditionally inactivated in the *Mef2c-AHF-Cre* lineage further affirms that the function of *Kmt2d* in the fourth PAAs is via interaction in the *Tbx1* lineage.

As fourth PAA formation is mediated via non-cell-autonomous signaling from the pharyngeal epithelia to mesenchymal cell types that include the SHF and NCCs (57), it is likely that there is cooperation of *Tbx1* and *Kmt2d* in this tissue. The pharyngeal epithelia are composed of endoderm and ectoderm within the pharyngeal apparatus, which invaginate to form pouches and clefts, respectively. Morphological assessment of the cKO embryos at E9.5 and E10.5 supports developmental dysmorphism and/or hypoplasia of the third pharyngeal pouch and fourth pharyngeal arch. While overall *Tbx1* expression levels were unchanged, its patterning was notably aberrant in the pharyngeal arches of cKO embryos at E9.5, consistent with the structural anomalies observed at E10.5. Further, *Epcam* expression at E10.5 revealed proper pouch invagination but highlighted the diminished area occupied by the third pharyngeal pouch due to fourth pharyngeal arch hypoplasia. The diminutive third pharyngeal pouch likely contributes to the reduced expression of *Foxn1* and *Gcm2*, markers for specific domains of the pouch that correspond to precursors of the thymus and parathyroid glands that were ectopic and/or hypoplastic at later developmental stages. Interestingly, the right lobe of the thymus was more severely impacted than the left, consistent with reports that left-right asymmetry, which normally occurs during development, is particularly exaggerated in phenotypes affecting pharyngeal structures (58).

To elucidate the mechanism behind the defects of the cKO, we performed scRNA-seq of cHet versus cKO embryos at E9.5 to identify specific genes that could be implicated in the phenotypic presentation. However, instead of standout genes with dramatically altered fold changes, we discovered many DEGs with small but significant changes that centered around basic homeostatic pathways. Our analysis largely focused on the main tissues of interest for the role of *Tbx1* in the pharyngeal region, including the epithelium, endothelium, aSHF, and MLPs. Overwhelmingly the DEGs, which were mostly downregulated, fell into categories of genes involved in gene expression, cell cycle regulation, and signaling transduction. Rather than implicating specific tissue types as causative of defects, the data of the integrated replicates pointed to a global and diffuse effect of the *Kmt2d* cKO, suggesting that the interplay of *Kmt2d* and *Tbx1* is multifaceted. This has been shown in other models of impaired chromatin regulation, which cause subtle but global effects on overall gene expression (59, 60). Our comparison of the scRNA-seq datasets between *Kmt2d*- and *Tbx1*-conditional-null embryos in the *Tbx1* lineage further supports this, as we identified similar GO terms upon analysis of the overlapping DEGs across the aforementioned clusters. What was particularly compelling about this analysis, however, was not just the pathways that were shared between the 2 datasets, but also those that were unique to the *Tbx1* cKO. This identified genes relevant to focal adhesion in the epithelium, blood vessel morphogenesis in the endothelium, OFT morphogenesis in the aSHF, and known Tbx1-associated genes in the MLPs. Since the phenotypes of the 2 models impact the same systems but with different severity, identifying these DEGs specific to the *Tbx1* cKO could shed light on genes that are most crucial in driving phenotypic presentation.

**Figure 10:**
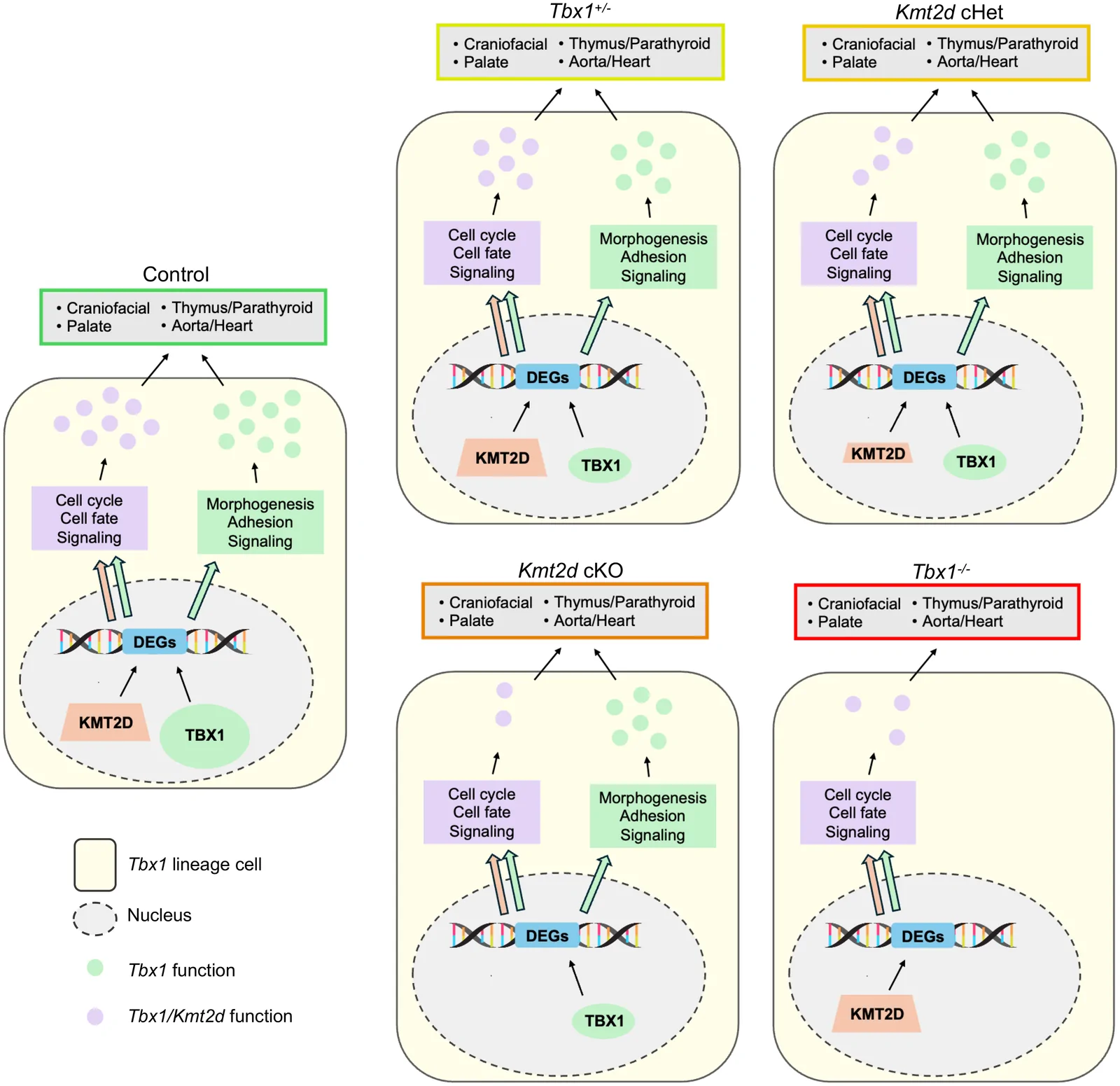
Molecular summary of *Kmt2d* and *Tbx1* interactions in mouse embryos. Model of how reduced gene dosage of *Tbx1* and/or *Kmt2d* impacts downstream pathways involved in embryonic development.

We depict a potential interplay of *Kmt2d* and *Tbx1* in Figure 10. In our previous TBX1 ChIP-seq and ATAC-seq studies, we found that TBX1 largely binds and opens chromatin at intergenic regions that include putative enhancers (21). TBX1 has also been shown to positively regulate H3K4 monomethylation and directly interact with KMT2C (61), suggesting that cooperation between KMT2D and TBX1 is likely localized to enhancer regions, allowing for recruitment of transcriptional machinery for the activation of downstream genes. Thus, reduced dosage of either gene leads to increased phenotypic severity. This is supported by our scRNA-seq data, as prominent downregulated DEGs across the different cell types were related to transcriptional machinery known to interact with KMT2D (10, 62). Furthermore, *Tbx1* functions with many of these complexes in its role as a transcription factor and has been shown to directly interact with some of them in early development (8, 63). In the same vein, we saw downregulation of genes involved in translational processes that are known to be targets of chromatin regulators in different developmental contexts, such as how *KMT2D* has been shown to be important for ribosomal protein regulation (64, 65). Finally, cell cycle/proliferation genes were downregulated, which is interesting since studies on *Kmt2d* pathogenesis in Kabuki syndrome show that its loss leads to a decrease in cell proliferation and cell cycle activity (66, 67).

Thus, the phenotype in *Kmt2d*-cKO embryos resembles an intermediate between *Tbx1*-heterozygous and -null mouse models, both phenotypically and genotypically. The defects observed in the P0 cKO pups result from abnormal pharyngeal arch morphology in early development and resemble other mouse models of 22q11.2DS. In this way, conditional inactivation of *Kmt2d* acts to provoke the developmental landscape of the *Tbx1* heterozygote, which otherwise has un-noteworthy phenotypes in a mixed background, to present with mild but lethal defects. This mirrors the data from the human study, which shows that variations in chromatin regulatory genes, including *KMT2D*, can explain severe cardiac presentations in patients with 22q11.2DS/*TBX1* haploinsufficiency. It also highlights notable similarities in the clinical presentations of 22q11.2DS and Kabuki syndrome, emphasizing the importance of expanding gene and protein networks to help clarify genotype/phenotype correlations. Ultimately, the identification of genetic modifiers of developmental pathology will allow us to further home in on the precision medicine needed to provide patients with improved diagnostic and therapeutic outcomes.

## Methods

### Sex as a biological variable.

Sex was not considered as a biological variable, as studies were conducted on embryos or newborn pups before morphological sexual characteristics can be determined.

### Mouse strains.

*Kmt2d^fl/fl^* mice (68), *Rosa26-Gfp^fl/fl^* mice (69), *Kmt2c^fl/fl^* (exon 3) mice (34), *Kmt2c^Δfl/fl^* (SET domain) mice (33), *Tbx1^Cre^* mice (26), *Mef2c-AHF-Cre* mice (70), and *Mesp1^Cre^* mice (71) have been previously described. All mice were backcrossed into a Swiss Webster background and then maintained in a mixed background (C57BL/6, Swiss Webster) unless otherwise described.

### qRT-PCR.

Cell preparation for qRT-PCR is described in Supplemental Methods. Total RNA was isolated from FACS-purified E9.5 embryos using the Arcturus PicoPure RNA Isolation Kit (Thermo Fisher Scientific, KIT0204). cDNA was generated using the SuperScript IV First-Strand Synthesis System (Thermo Fisher Scientific), and qRT-PCR reactions were performed in triplicate using the Power SYBR Green Master Mix (Applied Biosystems) and run on a 7900HT Real-Time PCR system (Applied Biosystems). Relative abundance of mRNAs was calculated by normalization to *Gapdh* mRNA levels. Primer sequences for *Gapdh* (72), *Tbx1* (73), and *Kmt2d* (25) have been previously described.

### Histology.

Embryos were generated from timed mating, and morning-time vaginal plug detection was considered as E0.5. For embryos at stage E11 or younger, somite count was used to confirm staging: E9.5 (23–28 somites), E10.5 (34–39 somites), E11 (40–45 somites). Embryos at stage E11.5 and earlier were collected in phosphate-buffered saline (PBS) and then fixed in 4% paraformaldehyde for 24–36 hours at 4°C. Embryos at E15.5 and above were fixed in 10% buffered formalin. After fixation, embryos were dehydrated through ethanol series (70%, 90%, 100%), cleared in xylene, and embedded in paraffin wax. The thickness of all paraffin sections was 10 μm, aside from whole embryos older than E15.5, which were sectioned at 12 μm. Hematoxylin and eosin (H&E) staining on histological sections was performed in the Molecular Cytogenetics Core at Albert Einstein College of Medicine using standard protocols.

### Immunofluorescence on cryosections.

Immunofluorescence on cryosections was performed as previously described (24) using primary antibody GFP (Abcam, ab6673; 1:500, goat) and secondary antibody Alexa Fluor 488 goat anti-mouse IgG (Invitrogen, A32723; 1:200). Images were taken using a Zeiss Axio Observer microscope with an apotome.

### Micro–computed tomography imaging and analysis of embryos.

Freshly collected E17.5 embryos and P0 neonates were fixed in 4% paraformaldehyde overnight at 4°C and transferred into PBS. In preparation for imaging, embryos and neonates were rinsed in 70% ethanol, air-dried for 5 minutes, and then placed on custom styrofoam beds that held the samples in a semi-upright posture. The specimens were then individually imaged using a Skyscan model 1275 micro–computed tomograph (micro-CT; Bruker BioSpin Corp.). Further imaging parameters and settings can be found in Supplemental Methods.

### Immunofluorescence on whole mount.

Whole-mount immunofluorescence was performed based on previous protocols (74) and as described in Supplemental Methods.

Primary antibodies included anti–β_III_-tubulin (Abcam, ab18207; 1:1,000), anti-CD31 (BD Biosciences, bd550274; 1:100), and anti-GFP (Abcam, ab6673; 1:100). Embryos were imaged using the Nikon CSU-W1 Spinning Disk confocal microscope from the Analytical Imaging Facility at Albert Einstein College of Medicine. Images were then processed using Imaris Viewer version 10.0.0 (Oxford Instruments, Abingdon, UK).

### India ink injection.

Fresh E10.5 embryos were dissected in PBS, and a solution containing 50% ink and 50% PBS was injected with India ink into the left ventricle using a microcapillary connected to an aspirator tube assembly. Imaging was performed using a Leica MX125 stereomicroscope.

### RNAscope on paraffin sections.

RNAscope in situ hybridization was performed on paraffin sections according to the manufacturer instructions (Advanced Cell Diagnostics, https://acdbio.com/ebook/introduction/rnascope-workflow) with standard tissue pretreatment recommendations and manual target retrieval. Briefly, E10.5 embryos were fixed in 4% paraformaldehyde at 4°C followed by ethanol dehydration and paraffin embedding. Sections of 8–10 μm were hybridized with RNAscope probes Mm-Tbx1 (Advanced Cell Diagnostics, 481911-C1), Mm-Foxn1 (Advanced Cell Diagnostics, 482021-C2), and Mm-Gcm2 (Advanced Cell Diagnostics, 530481-C3) using Multiplex Fluorescent Detection Reagents v2 (Advanced Cell Diagnostics, 323110). Detection of fluorescent signal was performed using tyramide signal amplification with Cy3, Cy5, and fluorescein, and sections were imaged using a Zeiss Axio Observer microscope with an apotome.

### Whole-mount RNAscope experiments.

RNAscope on whole-mount E9.5 embryos was performed as previously described (75) using Multiplex Fluorescent Detection Reagents v2 (Advanced Cell Diagnostics, 323110) and RNAscope probe Mm-Tbx1 (Advanced Cell Diagnostics, 481911-C1).

### Single-cell RNA-seq.

Single-cell sample preparation was performed as previously described (75) with further information in Supplemental Methods. Sequencing of the libraries was performed using an Illumina NovaSeq system (Azenta, South Plainfield, New Jersey, USA) with paired-end 150 bp read length.

### Single-cell RNA-seq analysis.

For each sample, the FASTQ files were processed using Cell Ranger version 8.0.1 to generate gene-by-cell count matrices. We then processed samples using scDAPP software version 1.2.0 (76), which integrates Seurat software version 5.1.0 (77) for individual clustering with RISC software version 1.7. Detailed analysis parameters and information can be found in Supplemental Methods. The final data were processed with ShinyCell (78) v1 for ease of community data sharing and can be accessed at https://scviewer.shinyapps.io/Kmt2d_and_Tbx1/

### Statistics.

Data were plotted using GraphPad Prism 10 software, and Student’s *t* test (unpaired, 2-tailed) or 3-way ANOVA was used to compare groups, with *P* < 0.05 considered significant.

### Study approval.

No data from human subjects were used in this study. All mouse studies were carried out according to NIH protocols approved by the Institutional Animal Care and Use Committee at Albert Einstein College of Medicine (protocol 00001034).

### Data availability.

All scRNA-seq datasets generated in this study were deposited in the NIH Gene Expression Omnibus (GEO) under the accession number GSE299068. All other data supporting the findings of this study are available within the article and its supplemental material, including the Supporting Data Values file, or upon request. Source data are provided with this paper.

## Author contributions

DM performed the mouse genetic studies, phenotypic analysis, and scRNA-seq and wrote the manuscript. KJ performed the scRNA-seq data analysis and generated relevant figures. TCC performed and analyzed the micro-CT scans. BEM led the project and edited the manuscript.

## Conflict of interest

The authors have declared that no conflict of interest exists.

## Funding support

This work is the result of NIH funding, in whole or in part, and is subject to the NIH Public Access Policy. Through acceptance of this federal funding, the NIH has been given a right to make the work publicly available in PubMed Central.

- NIH grants F30HL172604 to DM, F31HL180025 to KJ, R01HL157157 to BEM, and T32HL144456 and T32GM007288 to DM.
- Leducq Foundation 15CVD01 to BEM.
- Stowers Family Endowment for Dental & Musculoskeletal Tissue Research and the Langford Family Trust to TCC.

## Supplementary Material

Supplemental data

Supplemental table 1

Supporting data values

## Figures and Tables

**Table 1:**
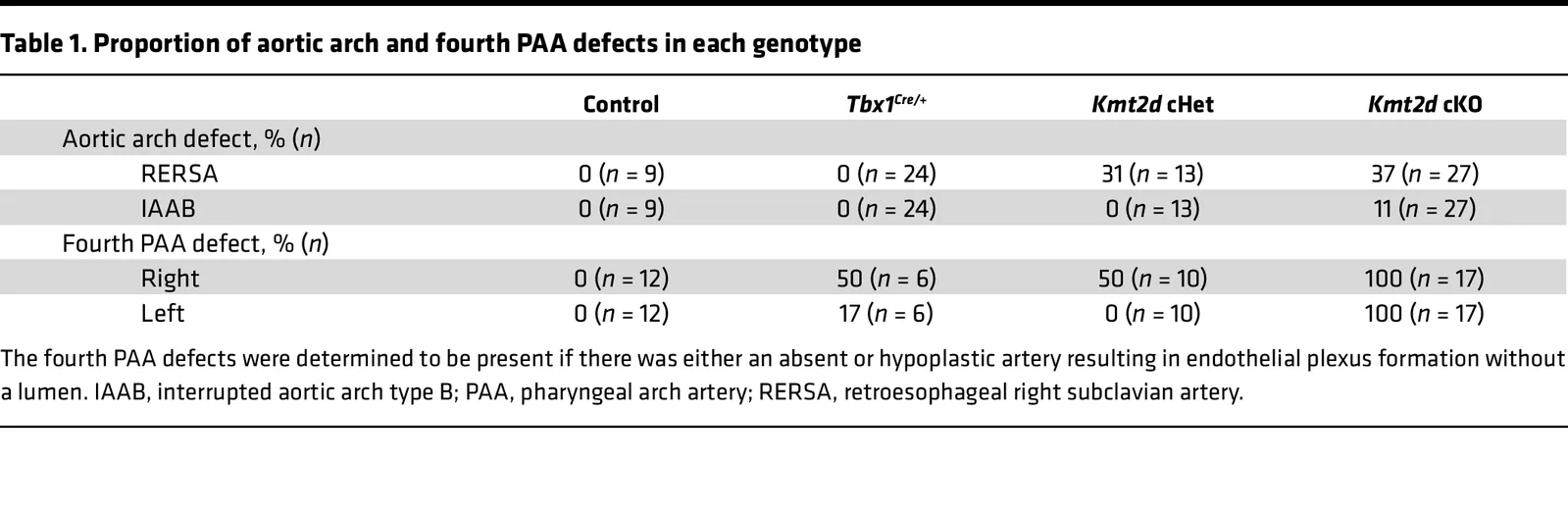
Proportion of aortic arch and fourth PAA defects in each genotype

**Table 2:**
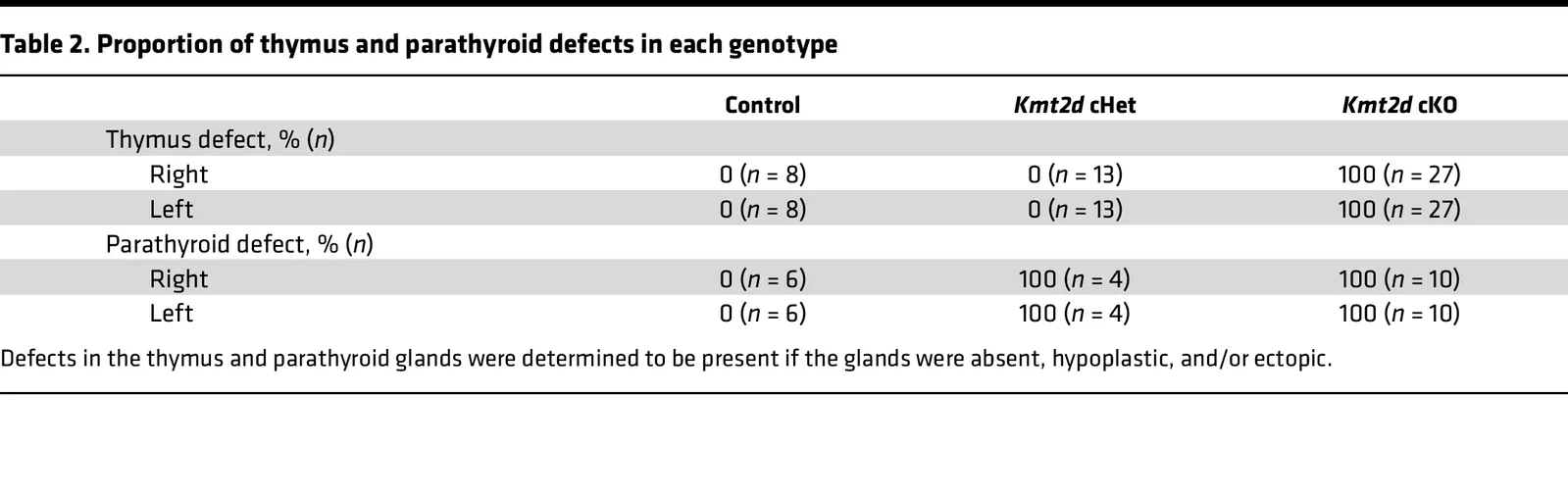
Proportion of thymus and parathyroid defects in each genotype

## Version 1 08/24/2026

Electronic publication
