# Supplementary material for: Reduced dosage of Kmt2d modifies Tbx1 haploinsufficiency toward phenotypes of 22q11.2DS
Source: JCI Insight. 2026 Aug 24;11(16):e206763. doi: 10.1172/jci.insight.206763 (PMC13502172; doi:10.1172/jci.insight.206763)
Supplement: Supplementary file 1 [file jciinsight-11-206763-s235.pdf]

Supplemental methods:

qRT-PCR (cont.)

E9.5 embryos were dissected and then incubated in 0.25% Trypsin-EDTA (Gibco, 25-200-056) together with DNase I (50 units/ml) for 8 min to dissociate tissue to a single cell suspension. Cells were FACS (Fluorescence Activated Cell Sorting) purified using a BD FACSAria II (BD Biosciences) system and BD FACSDiva 9.0.1 software in order to collect EGFP-positive and DAPI negative living cells in 1x PBS containing 10% FBS at 4°C. Following collection, these cells were centrifuged at 4 °C for 5 min at 300 × g and directly stored in −80°C freezer for short-term storage.

Microcomputed tomography imaging and analysis of embryos (cont.)

For imaging of soft tissue morphology and craniofacial skeleton in a single scan, the following parameters were used: 17.63 micron isotropic resolution, 40 kV, 200 microAmp, 45 ms exposure, 0.3° rotation step, 180° rotation, and 4-frame averaging. No filter was used. 16-bit raw images (tif format) from all scans were reconstructed to 8-bit bmp files using NRecon software v1.7.4.2 (Bruker BioSpin Corporation). The reconstructed scan datasets were then each imported into Drishti volume exploration software (version 2.63) (Limaye, 2012) for 3D rendering. Separate rendering settings were optimized for qualitative and quantitative phenotypic assessment of both mineralized and soft tissues.

For the assessment of internal soft tissues, P0 neonates were subsequently stained in Lugol's iodine solution for at least one week and then re-scanned on the microCT using the following settings: 20 micron isotropic resolution, 55 kV, 180 microAmp, 65 ms exposure, 0.3° rotation

step, 360° rotation, and 4-frame averaging, with a 0.5mm aluminum filter. These scan data were similarly reconstructed and rendered in Drishti.

Any non-specimen extraneous signal in rendered scan data that remained after optimizing the greyscale threshold was removed using one or more of the following tools within the Drishti program: 1) multiple clipping planes, 2) the morphological operations (MOP) carve function, and 3) the MOP erode/dilate functions. Selected images from the renderings were captured within Drishti, and subsequently further optimized for contrast, color, and background using Adobe Photoshop.

For assessment of soft tissue facial features, landmark-based quantitative measurements were taken directly in the rendered scans in Drishti. Specific inter-landmark distances served as proxies for the following anatomical measurements: head width, face width, midface length, lip width, mandibular length and width, interocular width, nasal tip width, medial upper lip (philtrum) height, pinna length. General craniofacial and skeletal features were also visually assessed through the use of distinct rendering settings in Drishti, and any differences from wildtype controls were noted.

Immunofluorescence on whole mount (cont.)

Briefly, embryos were dissected at E10.5 and fixed overnight in 4% PFA. After washing with PBS, embryos were incubated overnight in 0.1% PBS/100x Triton (PBS-T) and subsequently blocked overnight in 5% donkey serum. Embryos were then incubated with primary antibody in blocking buffer for 3-4 days, followed by an overnight wash in PBS-T and 4 x 1 hour washes the following day. This is followed by incubation in secondary antibody and DAPI (Thermo Scientific, #62248, 1:1000) for 2-3 days. Finally, embryos were washed as above and

progressively dehydrated in methanol before being cleared in BABB (1:2 mix of benzyl alcohol and benzyl benzoate). All steps were performed at 4°C aside from the final dehydration.

Single-cell RNA sequencing (cont.)

Embryos were collected at E9.5 and microdissected in 1x PBS at 4 °C. Dissected tissue was maintained in DMEM (Dulbecco's Modified Eagle Medium, GIBCO 11885084) at 4°C while genotyping was performed. Microdissections included the pharyngeal region from PA1 onwards and included the heart. Embryos were pooled by genotype and then incubated in 1 ml of 0.25% Trypsin-EDTA (GIBCO, 25200056) containing 50 U/ml of DNase I (Millipore, 260913-10MU) at room temperature for 7 min with occasional pipetting to break up the tissue. 100 uL of FBS (fetal bovine serum, ATCC, 30-2021) kept at 4°C was added to stop the reaction at a final concentration of 10%. Dissociated cells were centrifugated for 5 min at 300 × g at 4 °C and the supernatant was removed. Cells were then resuspended in 1x PBS (without Ca2+ and Mg2+, Coming, 21-031-cv) containing 10% FBS at 4 °C. These preparations were then filtered with a 100-μm cell strainer and 1 μL DAPI (1 mM) (Thermo Fisher Scientific, D3571) was added to each sample. Cells were FACS (Fluorescence Activated Cell Sorting) purified using a BD FACSAria II (BD Biosciences) system and BD FACSDiva 9.0.1 software in order to collect EGFP-positive and DAPI negative living cells in 1x PBS containing 10% FBS at 4°C. Following collection, these cells were centrifuged at 4 °C for 5 min at 300 × g. Finally, supernatant was removed and the cells were resuspended in 50 μL of 10% FBS for loading onto a 10x Chromium Instrument (10x Genomics). For Rep1 we used the Chromium Next GEM Single Cell 3′ Kit v3.1 (PN-1000268) and the Dual Index Kit TT Set A (PN-1000215). For Rep2 we used the Chromium GEM-X Single Cell 3′ Kit v4.1 (PN-1000691) and the Dual Index Kit TT Set A (PN-1000215).

Each scRNAseq sample sent for sequencing included a pool of 3 or more embryos of the same genotype.

Single cell RNA-seq analysis (cont.)

We ran the analysis with both pseudobulk mode “off” for individual replicates, and “on” for the combined replicates and used standard parameters, with the exception of min_num_Feature = 3000 and a resolution of 0.8. After identifying cell types of interest, the cells barcodes for a given population were then pulled and the raw data for these cells were then resubjected to scDAPP to ensure proper integration and mitigate batch effects Differentially expressed genes were calculated with the MAST test and filtered with the following parameters: For downregulated genes: FDR < 0.05, Log2FC < -0.25, Pct.2 (Control) > 0.1 (or 10% of cells). For upregulated genes: FDR < 0.05, Log2FC > 0.25, Pct.1 (KO) > 0.1 (or 10% of cells). Analysis of the DEGs was performed using a combination of ToppFun (1), the STRING database (2), and the Gene Set AI web portal (3).

References:

1. Chen J, Xu H, Aronow BJ, and Jegga AG. Improved human disease candidate gene prioritization using mouse phenotype. *BMC Bioinformatics.* 2007;8(1):392.
2. Szklarczyk D, Kirsch R, Koutrouli M, Nastou K, Mehryary F, Hachilif R, et al. The STRING database in 2023: protein–protein association networks and functional enrichment analyses for any sequenced genome of interest. *Nucleic Acids Research.* 2023;51(D1):D638-D46.
3. Hu M, Alkhairy S, Lee I, Pillich RT, Fong D, Smith K, et al. Evaluation of large language models for discovery of gene set function. *Nature Methods.* 2025;22(1):82-91.

Supplemental Figure 1

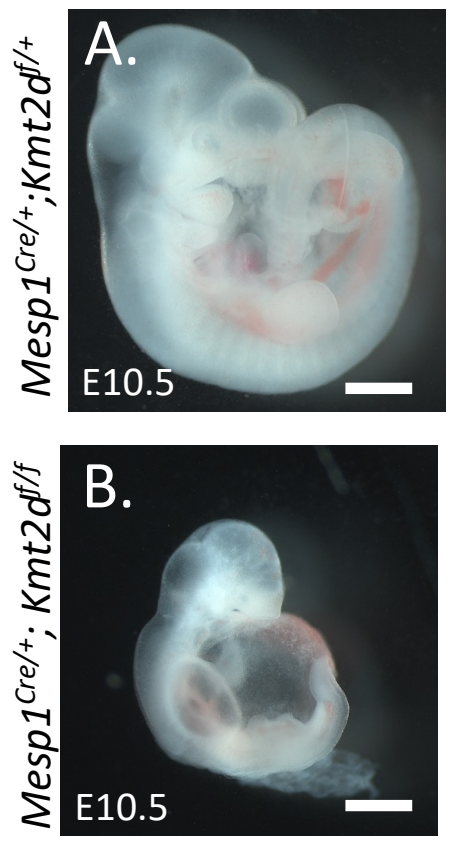

C.

Genetic Cross	Stage	# Litters	Total # Animals	# Mutants	% Mutant
$Mesp1^{Cre/+};Kmt2d^{f/+}$ X $Kmt2d^{f/f};Gfp^{f/f}$	E9.5	2	27	7	26
	E10.5	2	22	4	18
	E13.5	1	14	0	0
	E15.5	1	10	0	0
	P0	5	36	0	0

D.

	cHet (SW)	Control (SW)
Total # Animals	68	66
Expected Ratio	50%	50%
Observed Ratio	51%	49%

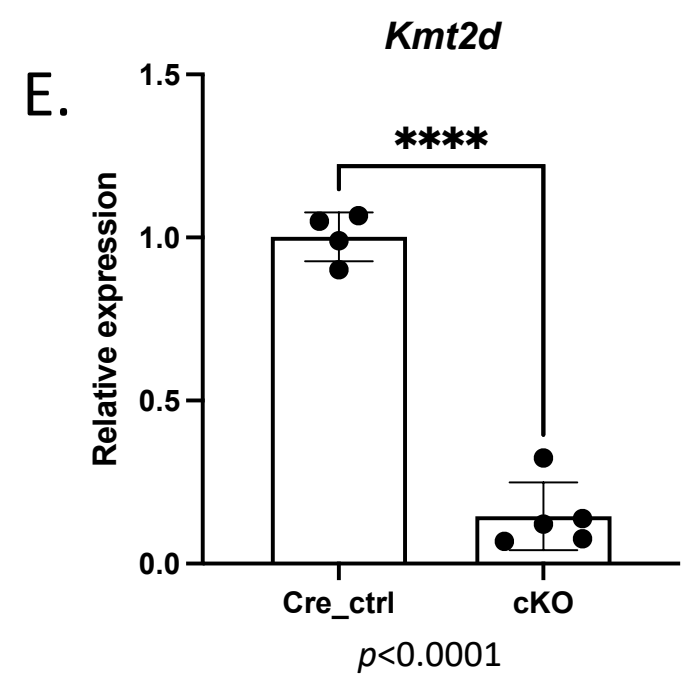

F.

Mouse Cross	Stage	# Litters	Total # Animals	# Mutants	% mutant
$Tbx1^{Cre/+};Kmt2d^{f/+}$ x $Kmt2d^{f/f};Gfp^{f/f}$	E9.5	2	18	4	22
	E11.5	4	35	9	26
	E12.5	2	18	6	33
	E16.5	3	20	5	25
	E17.5	9	107	31	29
	P0	14	139	31	22
	P1	14	139	0	0
	P8	26	180	0	0

Supplemental Figure 1: Validation of lethality of *$Mesp1^{Cre/+};Kmt2d^{f/f}$* embryos and mendelian ratios of *Kmt2d* cKO embryos with *$Tbx1^{Cre}$* and pups. Inactivation of floxed alleles of *Kmt2d* using *$Mesp1^{Cre/+}$* recapitulates the gross phenotypes and stage of lethality in a previously published report (PMID: 26932671). (A) Control *$Mesp1^{Cre/+};Kmt2d^{f/+}$* embryos at E10.5 display normal morphology. (B) Inactivation of both floxed alleles of *Kmt2d* using *$Mesp1^{Cre/+}$* leads to pericardial effusion in conditional null embryos by E10.5. (C) Table showing that these conditional null mutants (named 'Mutants') die by E10.5. (D) Table of

$Tbx1^{Cre/+}$;$Kmt2d^{f/+}$ (*Kmt2d* cHet) crosses in mixed genetic background (Swiss Webster, SW), showing no lethality in cHet embryos. (E) qRT-PCR for *Kmt2d* transcript levels in $Tbx1^{Cre/+}$ and cKO embryos. (F) Table of data from $Tbx1^{Cre/+}$ crosses to inactivate both alleles of *Kmt2d*, showing normal Mendelian ratios at various developmental stages and lethality of mutant embryos before P1. Scale bar: 250 μm.

Supplemental Figure 2

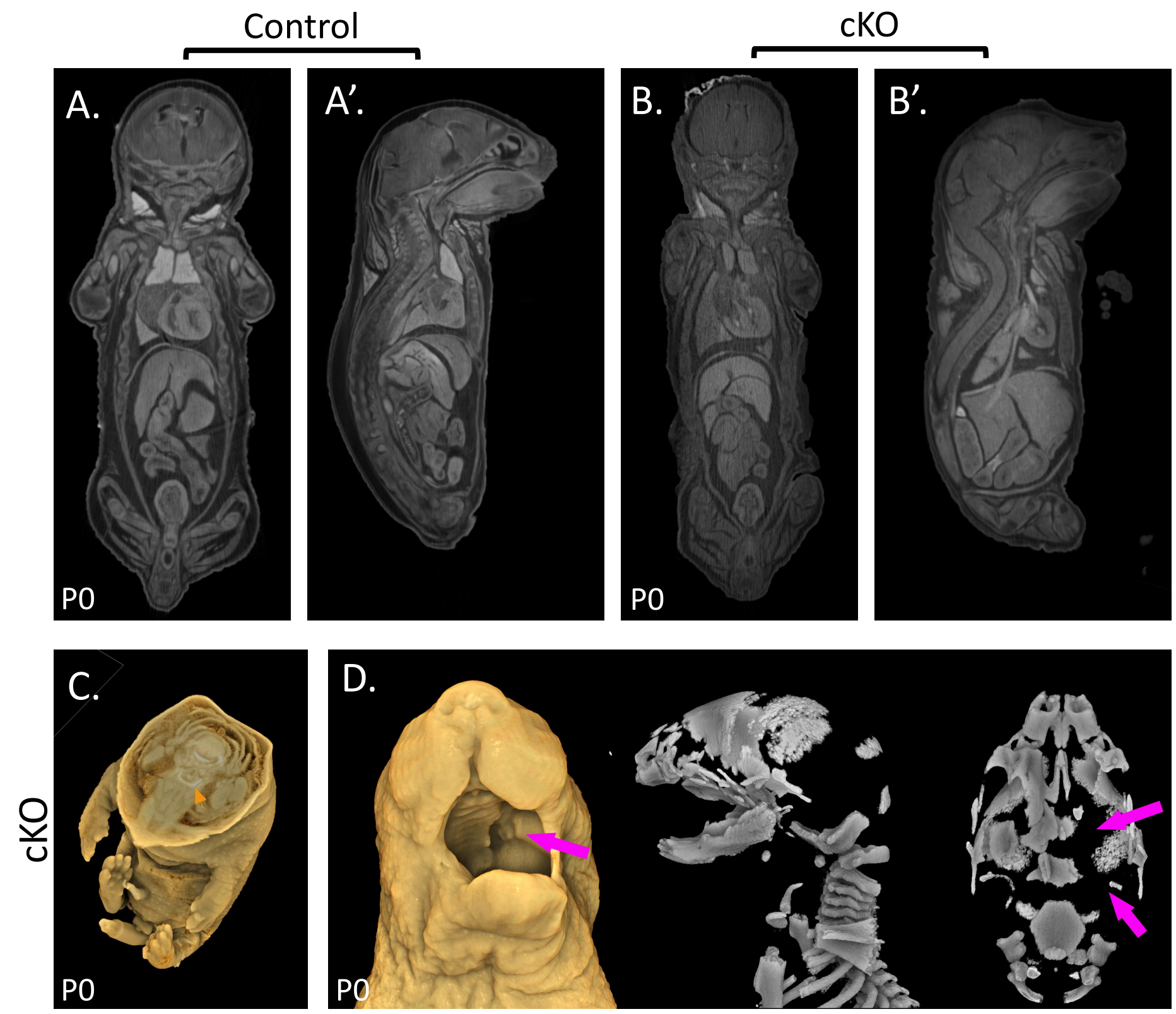

Supplemental Figure 2: Representative images of microCT scans of control and cKO neonates (P0). Representative images from iodine-stained microCT scans of neonates at P0 showing no gross organ defects in control (A) versus *Kmt2d* cKO (B) pups (n=3). (C) Representative cKO newborn mouse at P0 showing presence of the hyoid bone (orange arrow), which is absent in *Tbx1* null mouse embryos. (D) cKO newborn at P0 with a section of central bone (left palatal bone and left basal pterygoid of the basisphenoid) missing (pink arrowheads). This occurred in one P0 newborn mouse out of >20 examined.

Supplemental Figure 3

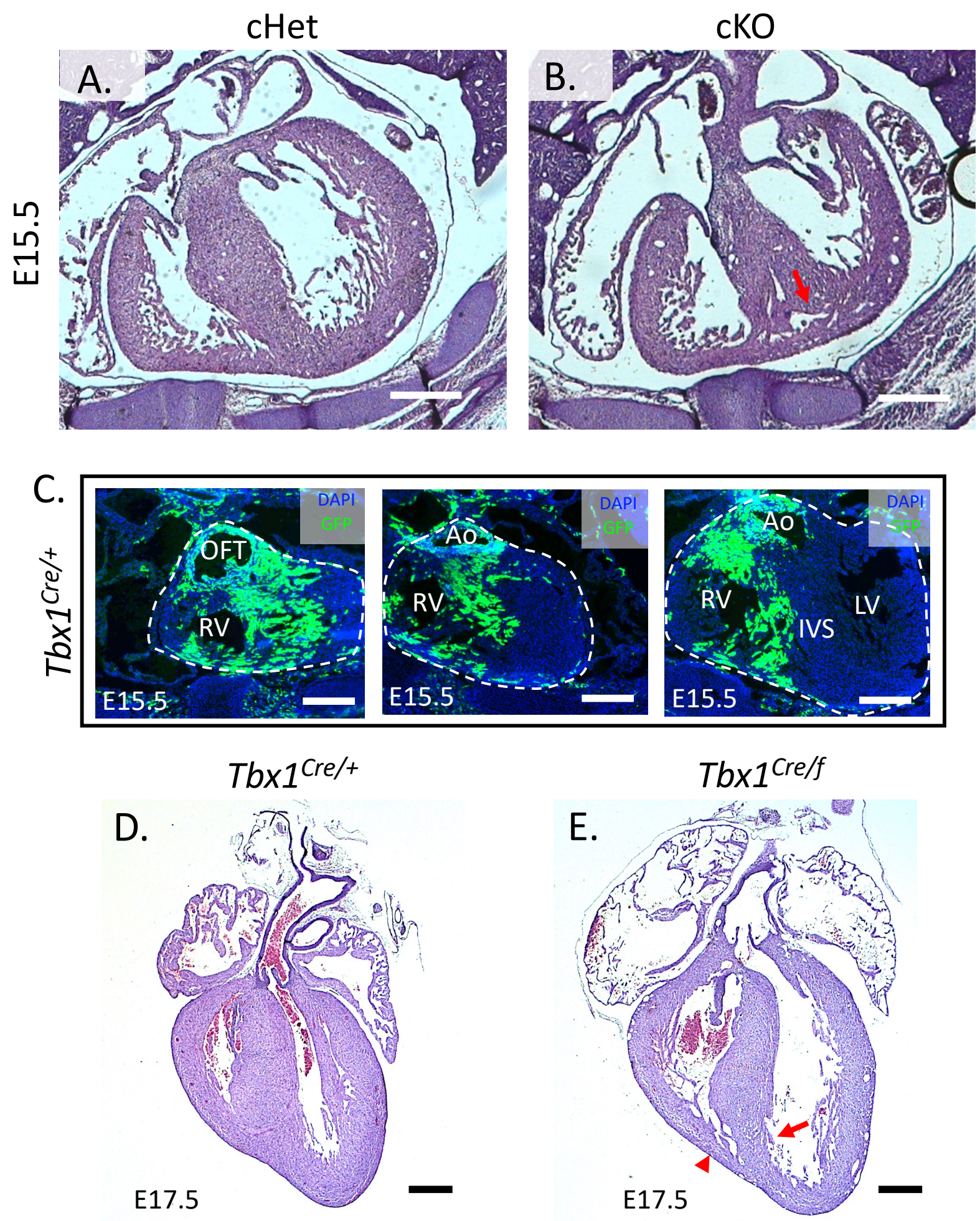

F.

Genotype	IVS Defects
Control	0 of 5
Tbx1 Cre/+	0 of 7
Tbx1 Cre/f	2 of 9
cKO	3 of 10

Supplemental Figure 3: Heart morphology in *Kmt2d* cKO and *Kmt2c;Kmt2d* double cKO embryos. (A) Transverse H&E stained section of the heart in cHet embryos at E15.5 showing normal morphology (n=3). (B) Transverse H&E stained section of cKO hearts at E15.5 showing disorganization of the interventricular septum (IVS) at the apex (red arrow; 3 of 10 cKO embryos showed this phenotype). (C) Serial transverse sections through an heart in *Tbx1*$^{Cre/+}$ embryos at E15.5 with immunofluorescence of GFP, showing the presence of *Tbx1* lineage in the RV

myocardium and adjacent IVS at the apex (n=2). (D) *Tbx1*$^{Cre/+}$ H&E stained section of an E17.5 heart showing normal morphology (n=7). (E) *Tbx1*$^{Cre/f}$ H&E stained section of a heart at E17.5 with abnormal morphology of the IVS (red arrow) and RV (red arrowhead) (n=2/9). (F) Table of the number of IVS defects found in each genotype. RV, right ventricle; LV, left ventricle; OFT, outflow tract; Ao, aorta; IVS, interventricular septum. Scale bar: 250 μm.

Supplemental Figure 4

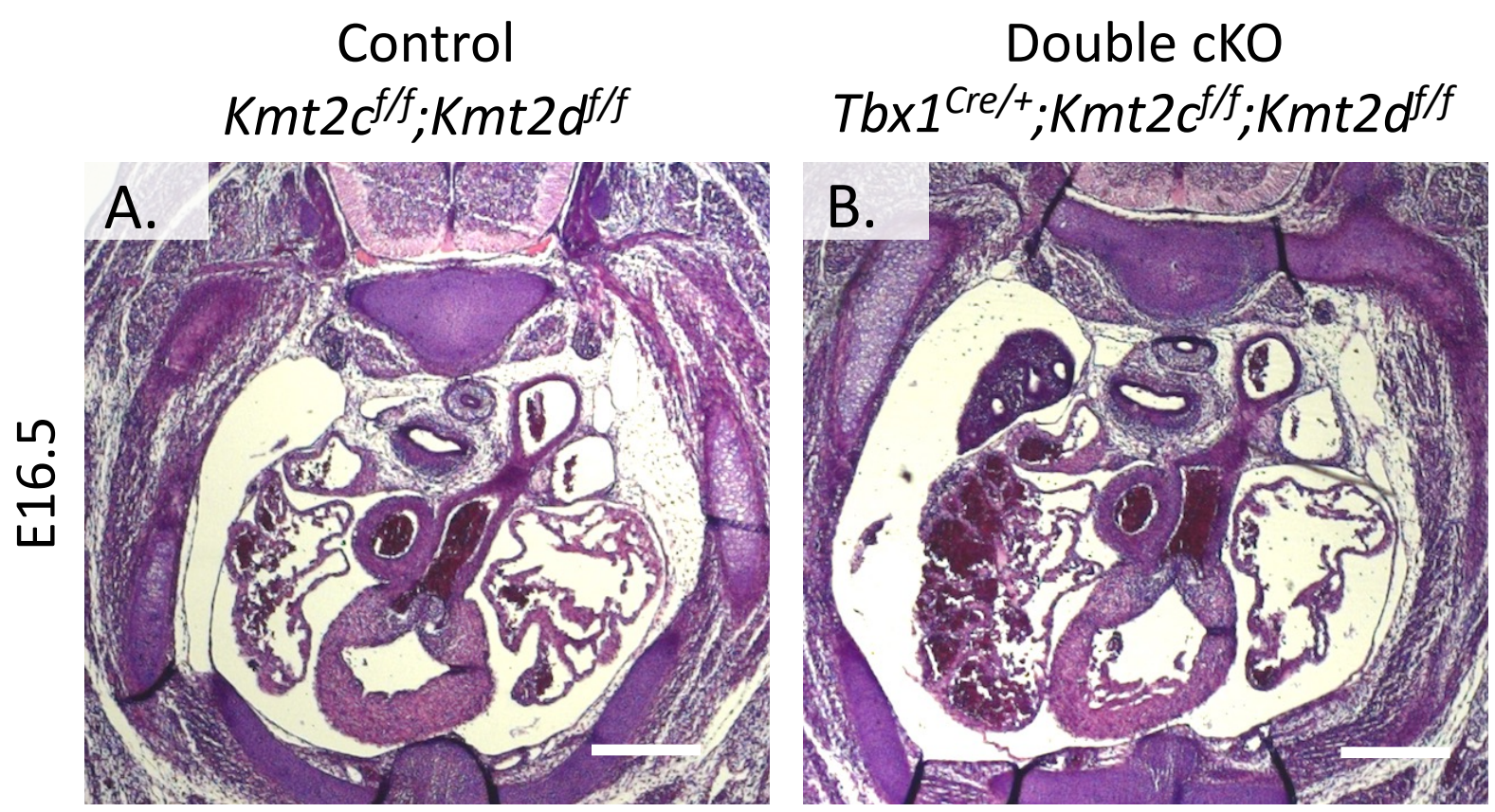

C.

Ratio of P21 Genotypes in *Tbx1 Cre Kmt2c/Kmt2d* Double cKO				
Tbx1 Cre/+	*Kmt2c*	*Kmt2d*	#	%
X	F/F	F/F	7	19.4
X	F/F	F/+	7	19.4
X	F/+	F/+	6	16.7
X	F/+	F/F	7	19.4
Cre/+	F/F	F/F	0	0
Cre/+	F/F	F/+	5	13.9
Cre/+	F/+	F/+	4	11.1
Cre/+	F/+	F/F	0	0

D.

Genetic Cross	Stage	# Litters	Total Animals	# Mutants	% Mutant
Tbx1$^{Cre/+}$;Kmt2c$^{\Delta f/+}$ x *Kmt2c$^{\Delta f/f}$;Gfp$^{f/f}$*	E15.5	4	33	4	12
	E16.5	2	19	4	21
	P0	4	28	4	14
	P8	4	16	5	31

Supplemental Figure 4: The *Kmt2c* and *Kmt2d* double mutant embryos do not show enhancement of defects compared to *Kmt2d* cKO mutant embryos alone. (A) Control *Kmt2d$^{f/f}$;Kmt2c$^{f/f}$* embryos and (B) double *Kmt2c;Kmt2d* cKO (*Tbx1$^{Cre/+}$;Kmt2d$^{f/f}$;Kmt2c$^{f/f}$*; n=5) embryos at E16.5, show proper septation of the aorta and pulmonary trunk (no occurrence of outflow tract defects). (C) Table of the genotypes identified in mice at weaning age (P21) in the double cKO cross (using the *Kmt2c* construct with deletion at exon 3). Only *Kmt2d* cKO mice exhibited lethality in the perinatal time period. (D) Table of the genotypes identified at P8 in a cross of *Kmt2c* (using the *Kmt2c* construct with deletion at the SET domain, Δ) conditional inactivation in the *Tbx1* lineage. No effect on viability was noted in any of the *Kmt2c* mutant offspring. Scale bar: 250 μm.

Supplemental Figure 5

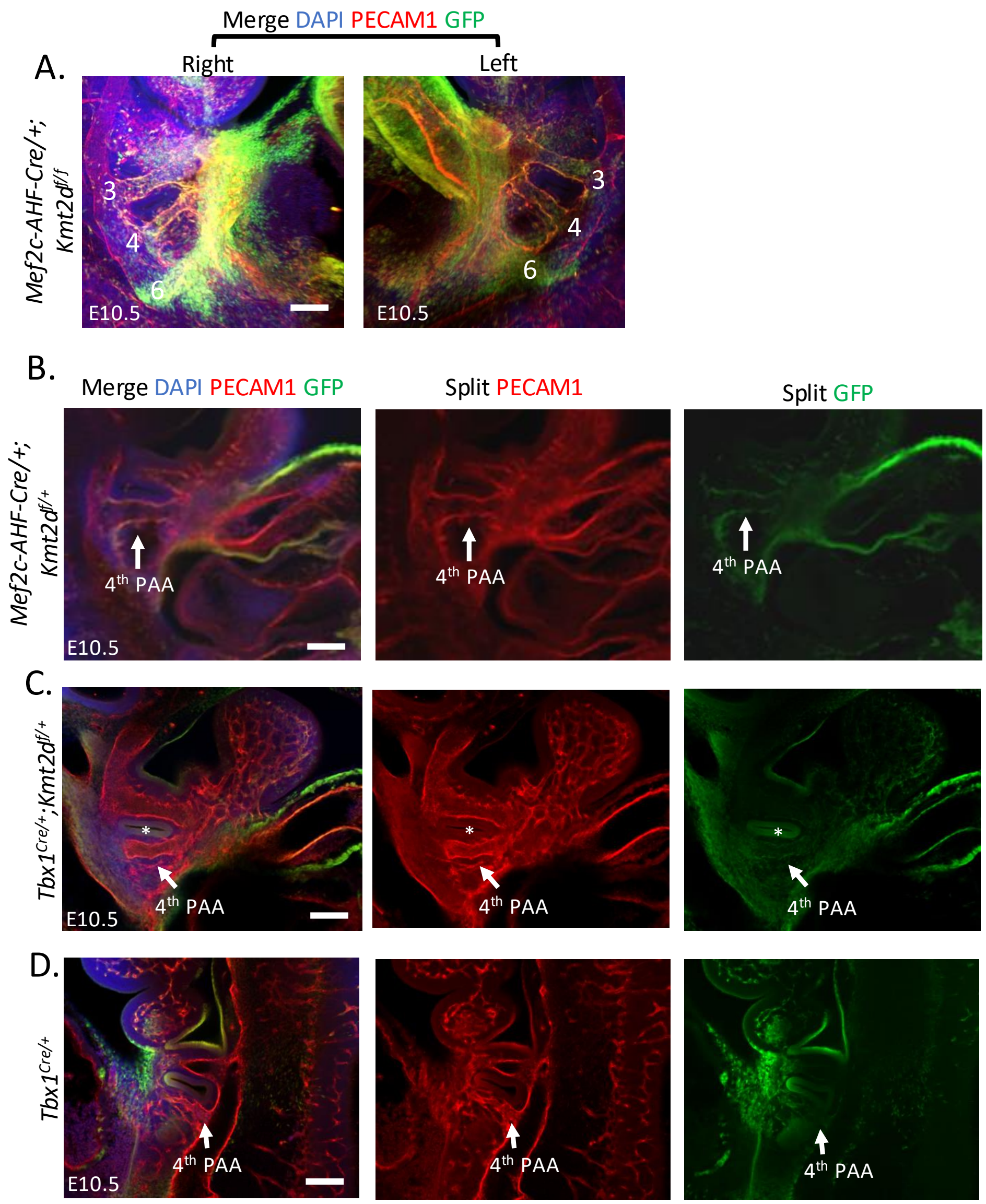

Supplemental Figure 5: Limited *Tbx1* lineage contribution to the 4th PAA endothelium versus that of *Mef2c-AHF-Cre* in embryos at E10.5. (A) *Mef2c-AHF-Cre/+;Kmt2d*$^{f/f}$ embryos have normal 4th PAAs (n=8). (B) *Mef2c-AHF-Cre/+;Kmt2d*$^{f/+}$ cHet embryos showing the *Mef2c-AHF-Cre* lineage in the 4th PAA by the presence of GFP overlapping with PECAM1 expression (n=5). (C) *Tbx1*$^{Cre/+}$;*Kmt2d*$^{f/+}$ cHet and (D) *Tbx1*$^{Cre/+}$ embryos showing limited cell lineage in the 4th PAA identified by the presence of GFP overlapping with PECAM1 (n=5 per genotype). Scale bar: 50 µm.

Supplemental Figure 6

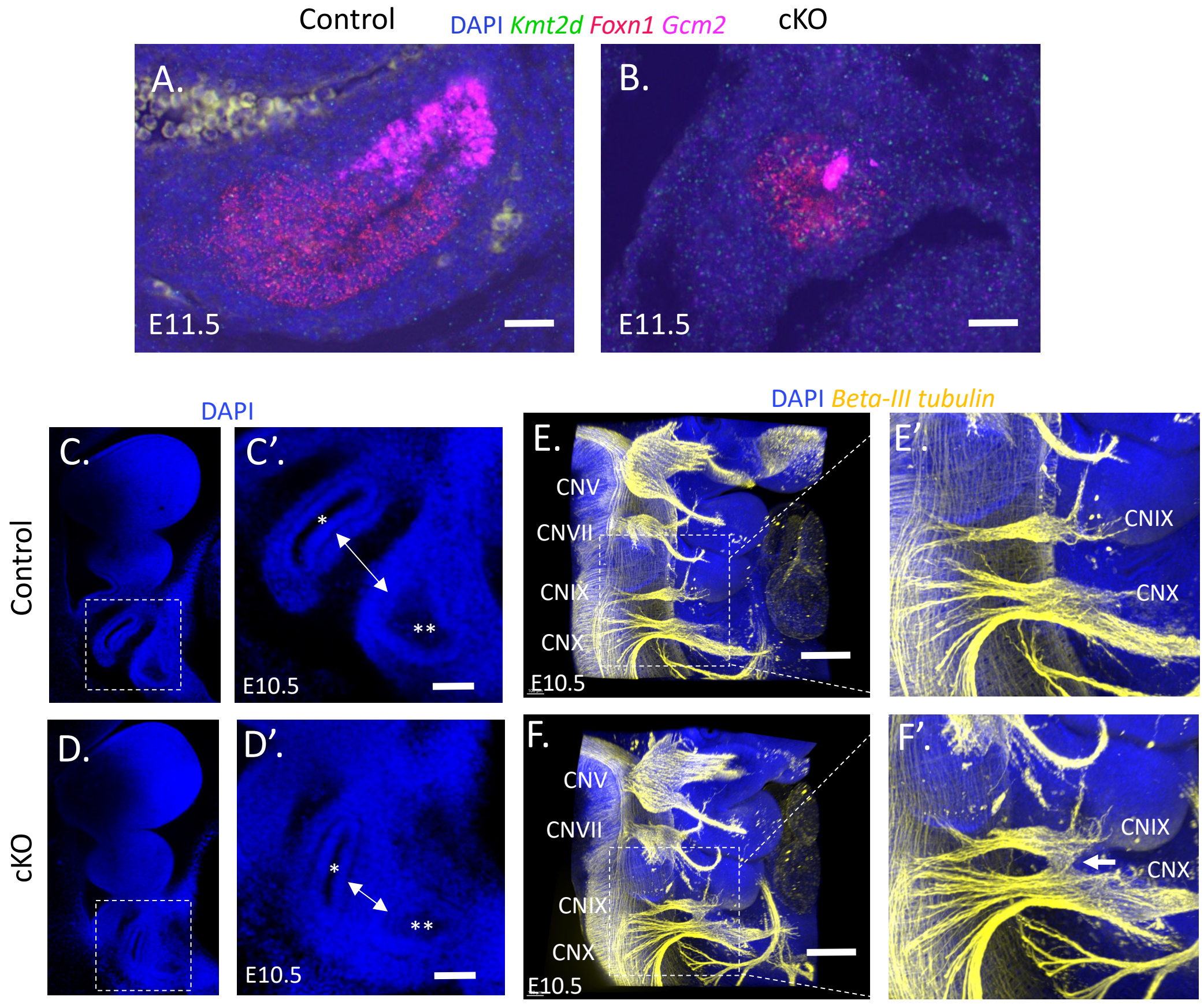

G.

	Cranial Nerve Defects	
**Genotype**	**Right**	**Left**
Control	20% (n=5)	40% (n=5)
cHet	40% (n=5)	60% (n=5)
cKO	50% (n=8)	88% (n=8)

Supplemental Figure 6: *Foxn1* and *Gcm2* expression in the 3rd pharyngeal pouch in additional replicates of control and *Kmt2d* cKO embryos at E11.5. (A) The third pharyngeal pouch is shown in a control (A) versus severely affected pharyngeal pouch in a *Kmt2d* cKO embryo (B). (A) Proper structure and localization of *Foxn1* (red) and *Gcm2* (pink) expression in the ventral and dorsal aspects of the pouch, respectively. (B) *Kmt2d* cKO embryo has hypoplasia of the third pharyngeal pouch and abnormal expression pattern of *Foxn1* and *Gcm2*. (C) Sagittal view of a control embryo showing normal 3rd (*) and 4th (**) pharyngeal pouch morphology which are separated by the 4th PAA. Double-sided arrow illustrates the distance between the pouches. (D) Sagittal view of a *Kmt2d* cKO embryo showing hypoplasia of the 3rd (*) and 4th (**) pharyngeal pouches with absent 4th PAA separation. Double-sided arrow illustrates decreased distance between the pouches in mutant embryos. (E) Representative cranial nerve (CN) staining visualization using beta-tubulin in control embryos at E10.5 showing presence and

proper patterning of cranial nerves V, VII, IX, and X. (F) Similar beta-tubulin staining of select *Kmt2d* cKO littermates at E10.5 showing presence of cranial nerves but fusion (arrow) and decreased distance between CNIX and CNX. (G) Table of replicates for cranial nerve analysis. Scale bar: (A-B) 50 μm (C-D) 15 μm (E-F) 200 μm.

Supplemental Figure 7

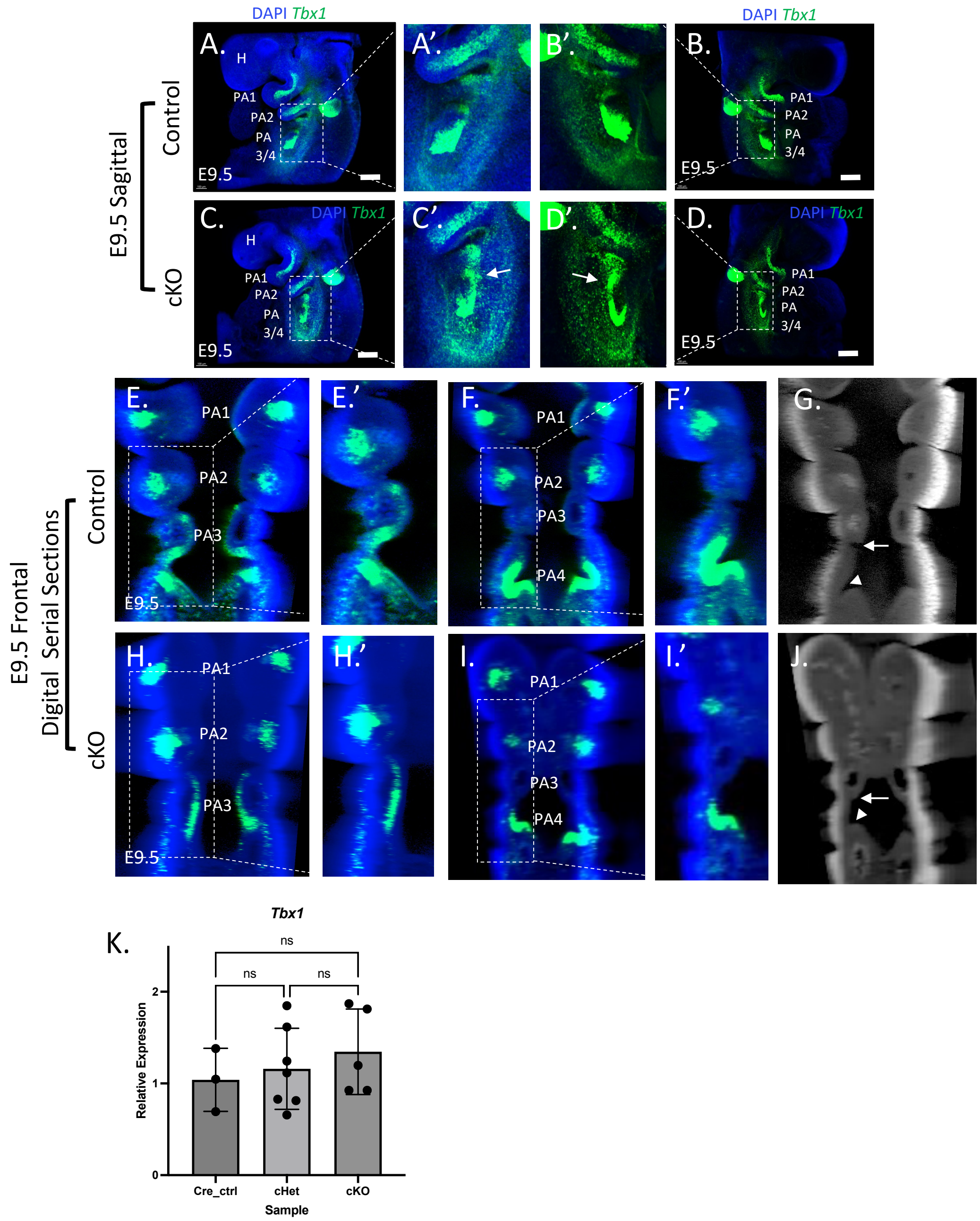

Supplemental Figure 7: Difference of the pharyngeal pouch, cranial nerve staining, and *Tbx1* expression in cKO versus controls. RNAscope analysis of *Tbx1* expression on the (A) left and (B) right side of control embryos and the (C) left and (D) right side of the cKO embryos at E9.5, showing abnormal expression of *Tbx1* in cKO embryos (n=7). (E-G) Digital images of the *Tbx1* expression to provide a frontal view of control embryos in (A-B). *Tbx1* expression is present in the 3rd pharyngeal pouch in (E) and the forming 4th pharyngeal pouch in (F). (G) Invagination can be seen in a DAPI (grey) frontal image, highlighting the 3rd (arrow) and 4th (arrowhead) pharyngeal pouches. (H-J) Digital images of the *Tbx1* expression to provide a

frontal view of cKO embryos in (C-D). This showcases improper expression of *Tbx1* or pharyngeal pouch structure in the region of the 3rd PA (H) and reduced expression in the 4th pharyngeal pouch in (I). A DAPI (grey) frontal image (J) reveals delayed/incomplete invagination of the 3rd (arrow) and 4th (arrowhead) pharyngeal pouches. (K) qRT-PCR for *Tbx1* transcript levels in $Tbx1^{Cre/+}$, cHet, and cKO embryos. Scale bar: 500 μm

Supplemental Figure 8

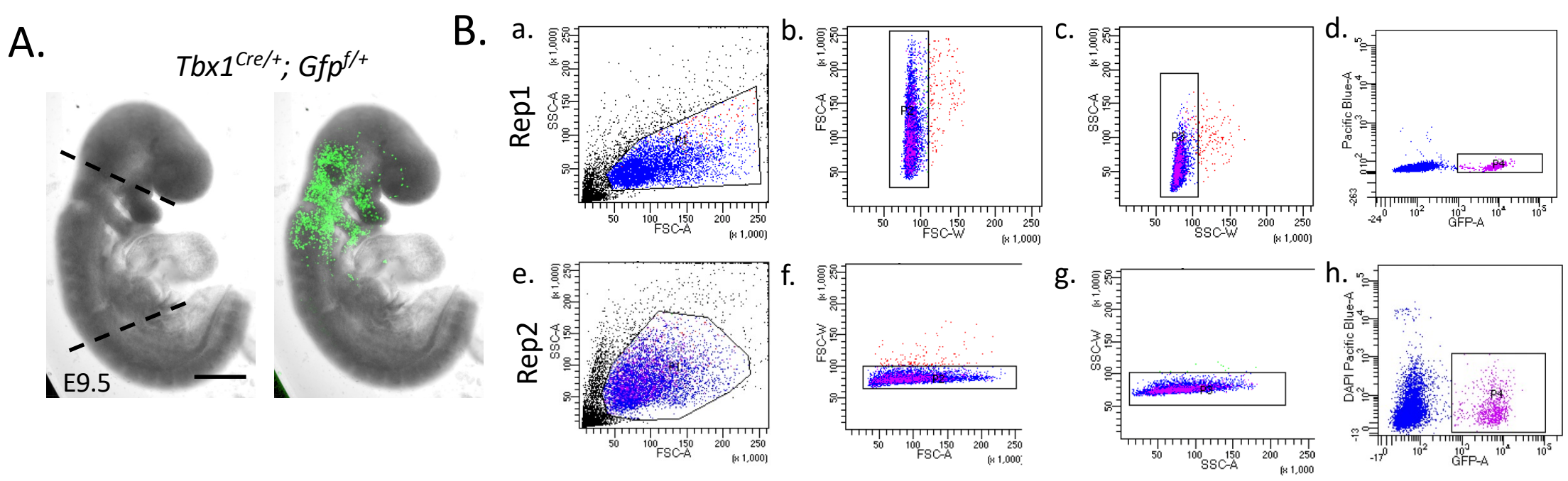

C.

Rep1 Sample	Genotype	# Embryos	Stage	# Somites	# FACS Purified Cells	Viability	Total Cells	Average Genes/ Cell	Average Reads/ Cell
Kmt2d cHet	$Tbx1^{Cre/+}$; $Kmt2d^{f/+}$; $Gfp^{f/+}$	4	E9.5	23-27	54066	87%	5918	4650	50050
Kmt2d cKO	$Tbx1^{Cre/+}$; $Kmt2d^{f/f}$; $Gfp^{f/+}$	4	E9.5	25-26	45930	85%	9307	4843	43970

Rep2 Sample	Genotype	# Embryos	Stage	# Somites	# FACS Purified Cells	Viability	Total Cells	Average Genes/ Cell	Average Reads/ Cell
Kmt2d cHet	$Tbx1^{Cre/+}$; $Kmt2d^{f/+}$; $Gfp^{f/+}$	3	E9.5	23-25	34497	81%	13192	5396	48072
Kmt2d cKO	$Tbx1^{Cre/+}$; $Kmt2d^{f/f}$; $Gfp^{f/+}$	3	E9.5	25-26	45266	77%	16775	4858	40016

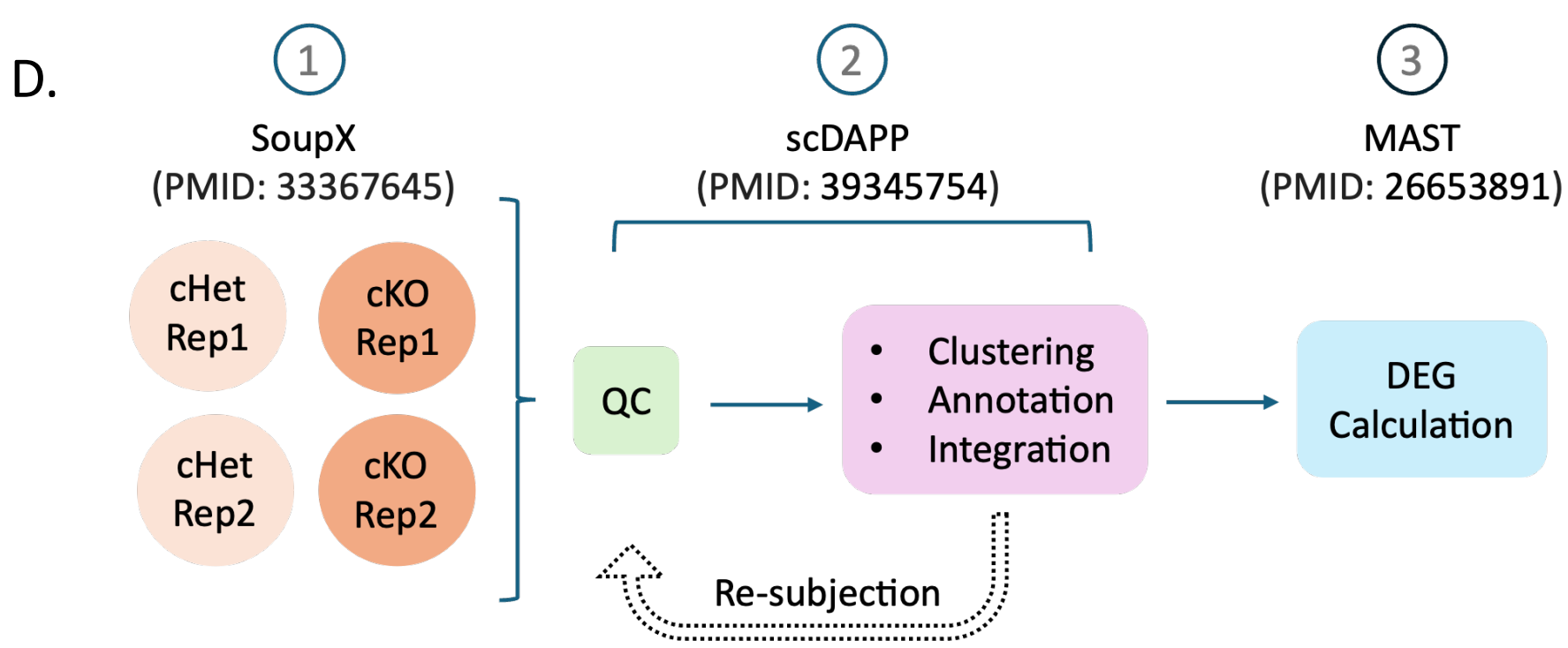

Supplemental Figure 8: Single cell RNA-seq experiment at E9.5 and pipeline of data analysis. (A) Representation of the embryos used for scRNAseq. Left: wild type embryo with dotted lines indicating the microdissection for scRNAseq just above the first pharyngeal arch to right below the heart. Right: $Tbx1^{Cre/+}$;$Gfp^{f/+}$ embryo with GFP marking the lineage that was sorted for using fluorescence activated cell sorting (FACS). (B) Gating strategy for GFP positive cell sorting for Rep1 (a-d) and Rep2 (e-h). a) FSC-A/SSC-A (FSC-A, forward scatter area; SSC-

A, side scatter area) plot show gated cells. B-c) Singlets were gated in FSC-W/FSC-A (FSC-width, FSC-area) plot (b) and in SSC-W/SSC-A (SSC-width; SSC-area) plot (c). d) GFP+ viable cells were gated in GFP-A/Pacific Blue-A plot. e) Table of total number of FACS purified GFP+ cells in each sample. (C) Tables of the information for the samples in the scRNAseq experiment for Rep1 and Rep2. (D) Flow diagram illustrating the workflow for the scRNAseq analysis. First, individual samples were processed with SoupX (PMID: 33367645) to remove ambient mRNA. Next, SoupX cleaned individual samples were subjected to the scDAPP pipeline (PMID: 39345754) for further QC, integration, and cell type annotation. This was done for Rep1, Rep2, Rep1/Rep2 combined, and the *Tbx1* cKO datasets. After identifying cell types of interest, cells from all samples for a given cell type were then subset and resubjected to scDAPP analysis. Differential expression was calculated using the MAST method. Scale bar: 500 μm.

Supplemental Figure 9

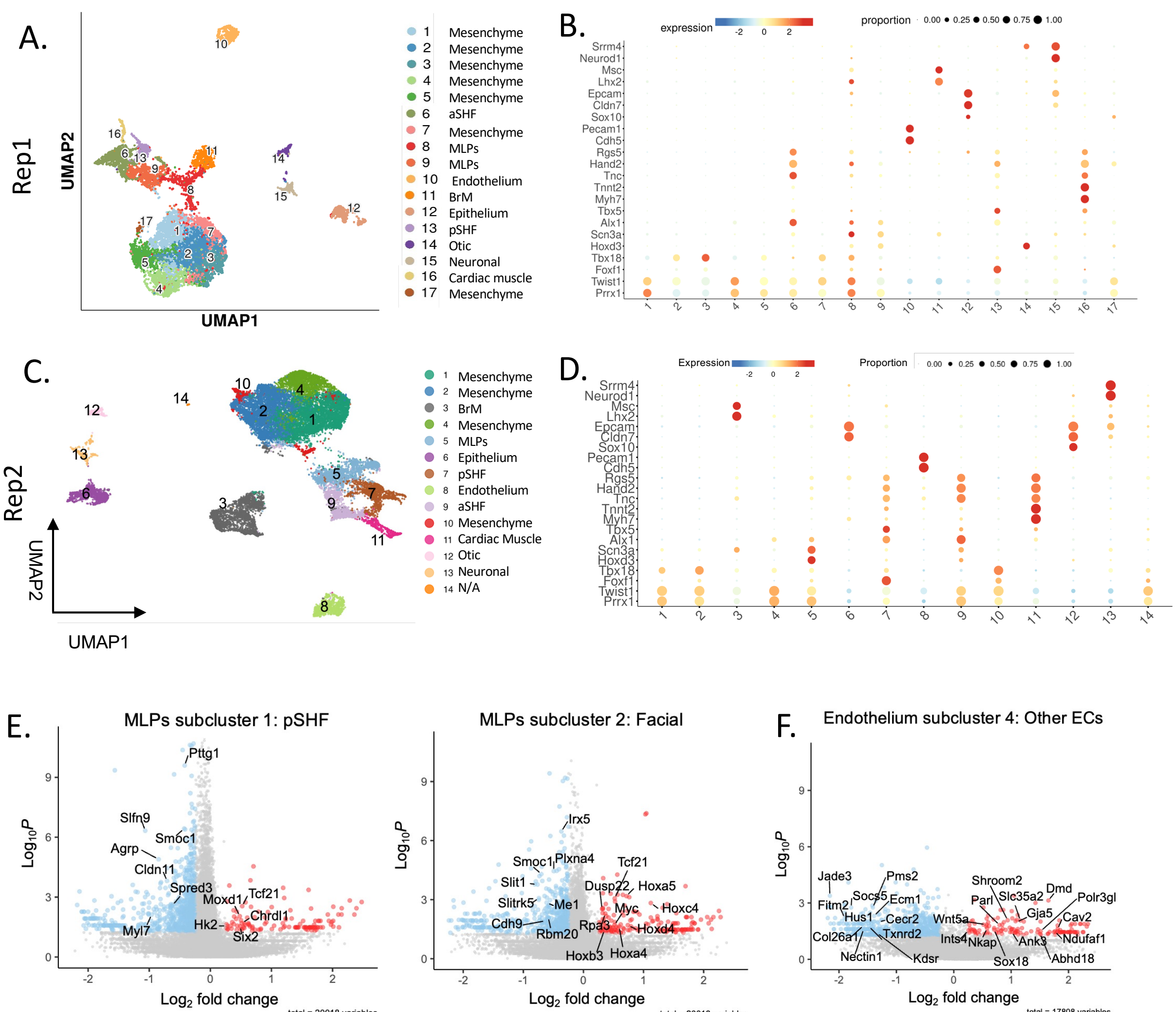

Supplemental Figure 9: Single cell RNA-seq experiment additional plots. (A) Integrated UMAP and (B) dot plot of the first scRNAseq replicate (Rep1). (C) Integrated UMAP and (D) dot plot of the second replicate for scRNAseq (Rep2). (E) Volcano plots for the remaining subclusters discussed in Fig. 7.

Supplemental Figure 10

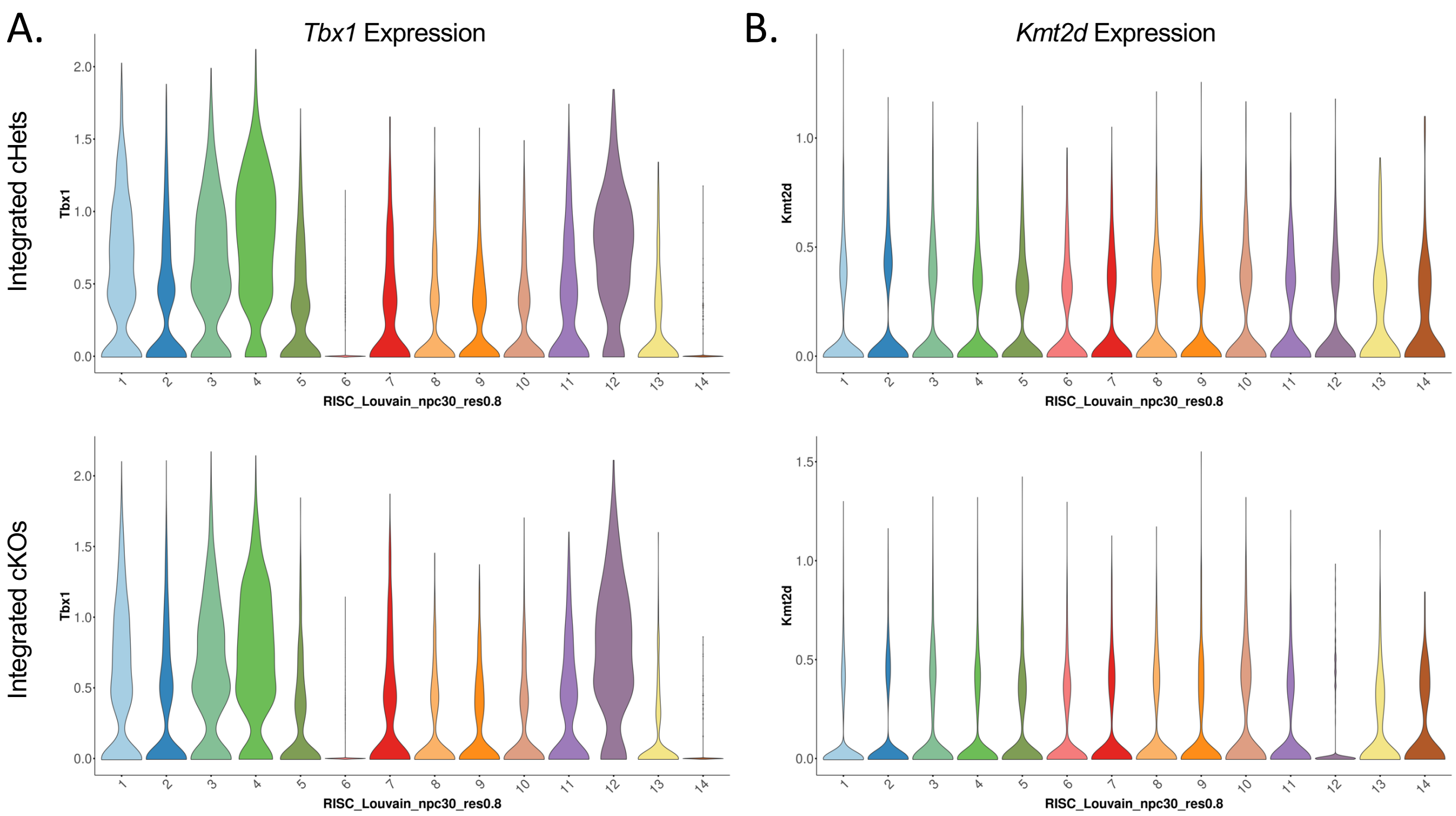

Supplemental Figure 10: Expression of *Tbx1* and *Kmt2d* at E9.5 from scRNAseq data of cHet and cKO replicates. (A) Violin plots showing the expression level of *Tbx1* in each cluster of the combined cHet replicates (top) compared to the combined cKO replicates (bottom). (B) Violin plots showing the expression level of *Kmt2d* in each cluster of the combined cHet replicates (top) compared to the combined cKO replicates (bottom).
